# Supplementary material for: LCO Receptors Involved in Arbuscular Mycorrhiza Are Functional for Rhizobia Perception in Legumes
Source: Curr Biol. 2019 Dec 16;29(24):4249–4259.e5. doi: 10.1016/j.cub.2019.11.038 (PMC6926482; doi:10.1016/j.cub.2019.11.038)
Supplement: Document S1. Figures S1–S7, Tables S1 and S2, and Reference S1, Related to Figures 1, 2, 3, 5, and 6 [file mmc1.pdf]

Current Biology, Volume *29*

Supplemental Information

LCO Receptors Involved in Arbuscular Mycorrhiza Are Functional for Rhizobia Perception in Legumes

Ariane Girardin, Tongming Wang, Yi Ding, Jean Keller, Luis Buendia, Mégane Gaston, Camille Ribeyre, Virginie Gasciolli, Marie-Christine Auriac, Tatiana Vernié, Abdelhafid Bendahmane, Martina Katharina Ried, Martin Parniske, Patrice Morel, Michiel Vandenbussche, Martine Schorderet, Didier Reinhardt, Pierre-Marc Delaux, Jean-Jacques Bono, and Benoit Lefebvre

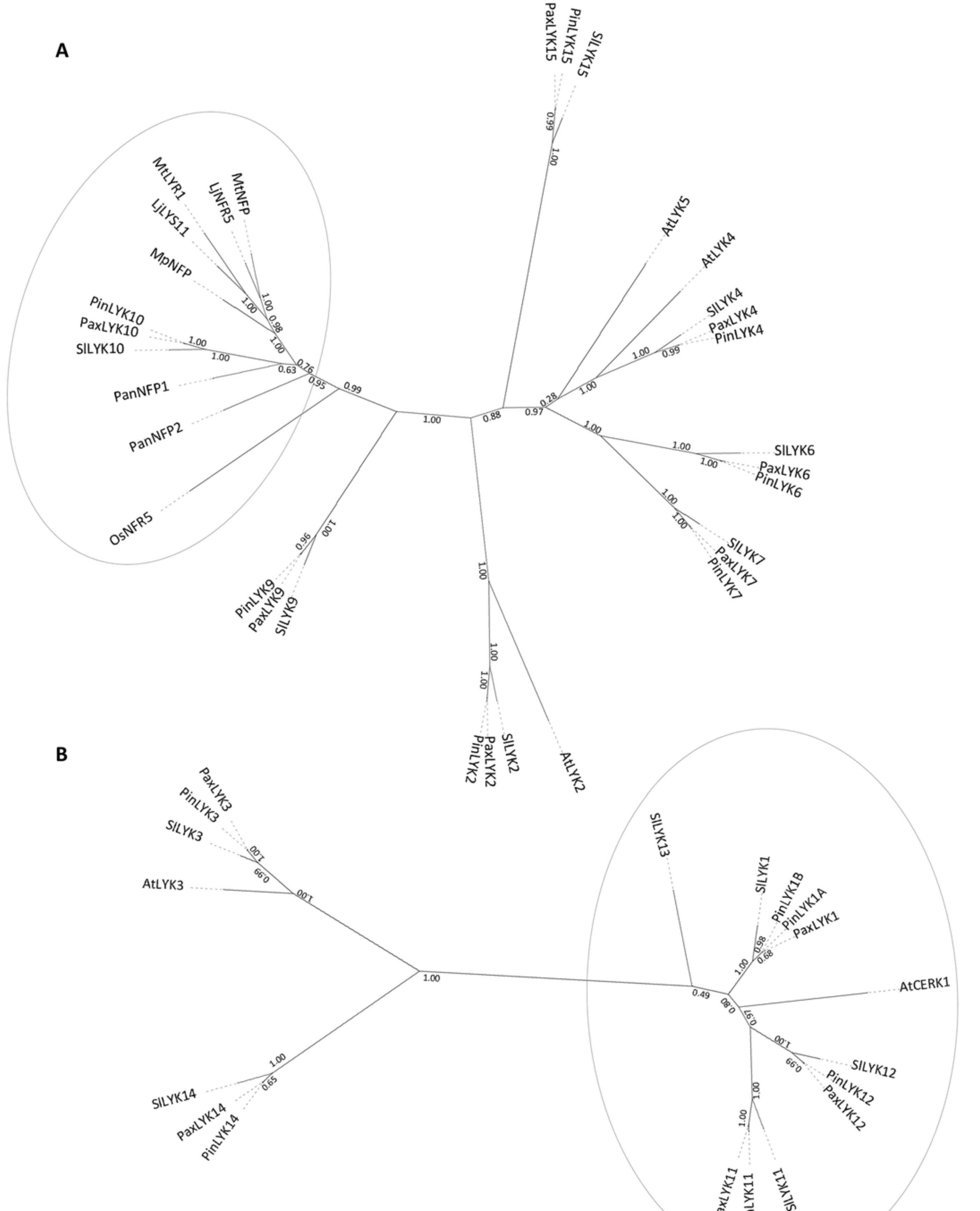

Figure S1 Phylogenetic trees of LYR and LYK LysM-RLKs. **Related to Figures 1 and 2.** Full length amino acid sequences of all LYRs, predicted to contain inactive kinase domains (A) or LYKs, predicted to contain active kinase domains (B) from *Petunia axillaris* (Pax), *Petunia inflata* (Pin), *Solanum lycopersicum* (Sl) and *Arabidopsis thaliana* (At) were used to construct phylogenetic trees. Accession numbers of *P. axillaris and P. inflata* LysM-RLKs can be found in table S1. Members of the phylogenetic group *LYRIA* (A, circled) from *Medicago truncatula* (Mt), *Lotus japonicus* (Lj), *Mimosa pudica* (Mp), *Parasponia andersonii* (Pan) and *Oryza sativa* (Os) were added. SlLYK8 was not included because it has a truncated kinase. The phylogenetic group *LYKI* containing AtCERK1 and SlLYK12 is circled (B). Numbers at the branches correspond to approximate likelihood values.

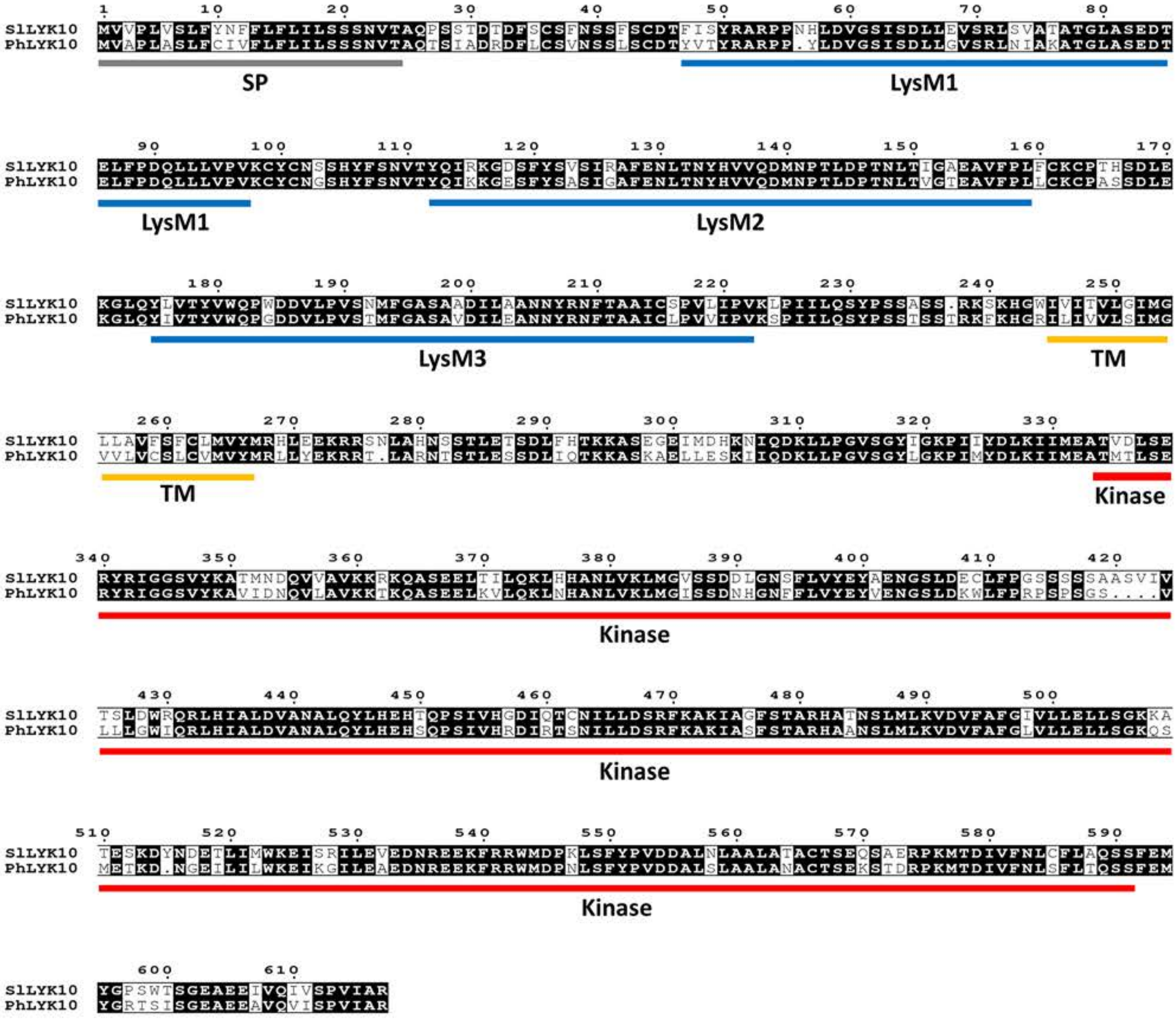

Figure S2 Sequence alignment and annotation of PhLYK10 and SlLYK10. Related to Figures 1 and 2. *PhLYK10* in *Petunia hybrida* originates from the *Petunia axillaris* parent. PhLYK10 has 79% amino acid identity compared to SlLYK10.

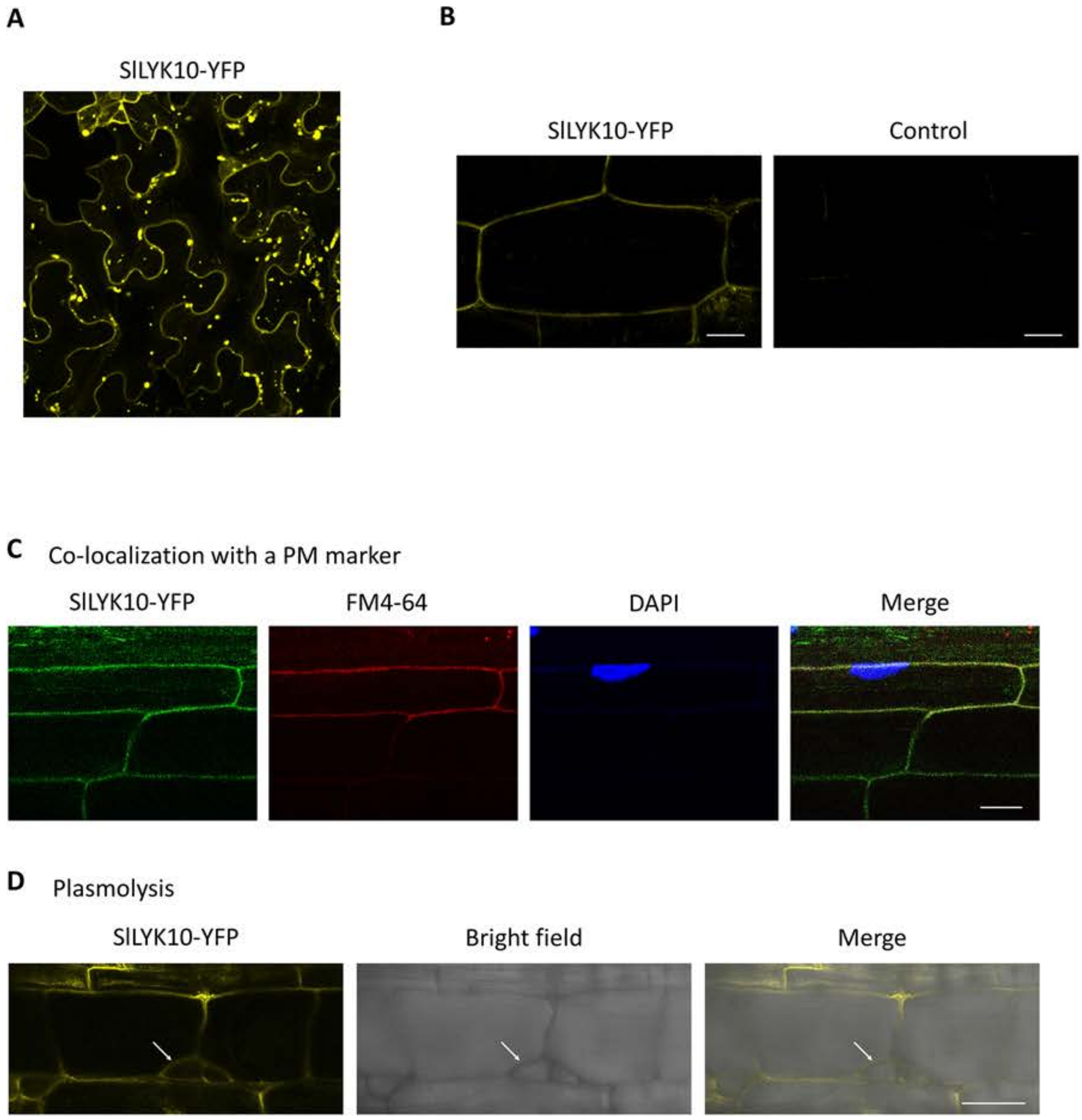

Figure S3 Subcellular localization of SlLYK10-YFP in *N. benthamiana* leaves and tomato roots. Related to Figure 3. A) Confocal images of epidermal cells from a *N. benthamiana* leaf expressing *Sl*LYK10-YFP and showing localization partly in undefined compartments. B) Confocal images of epidermal cells from a tomato ROC line expressing SlLYK10-YFP or a ROC control. Images were acquired with similar microscope settings. C) Confocal images of epidermal cells from a tomato ROC line expressing SlLYK10-YFP and treated with FM4-64 (which labels the plasma membrane (PM) before being internalized) and DAPI (which labels the nucleus), showing that SlLYK10-YFP is localized at the PM. D) Confocal images of epidermal cells from a tomato ROC line expressing SlLYK10-YFP and plasmolyzed with mannitol. The arrow points to detachment of the PM from the cell wall. Scale bars represent 20 µm in A, 50 µm in B and 10 µm in C.

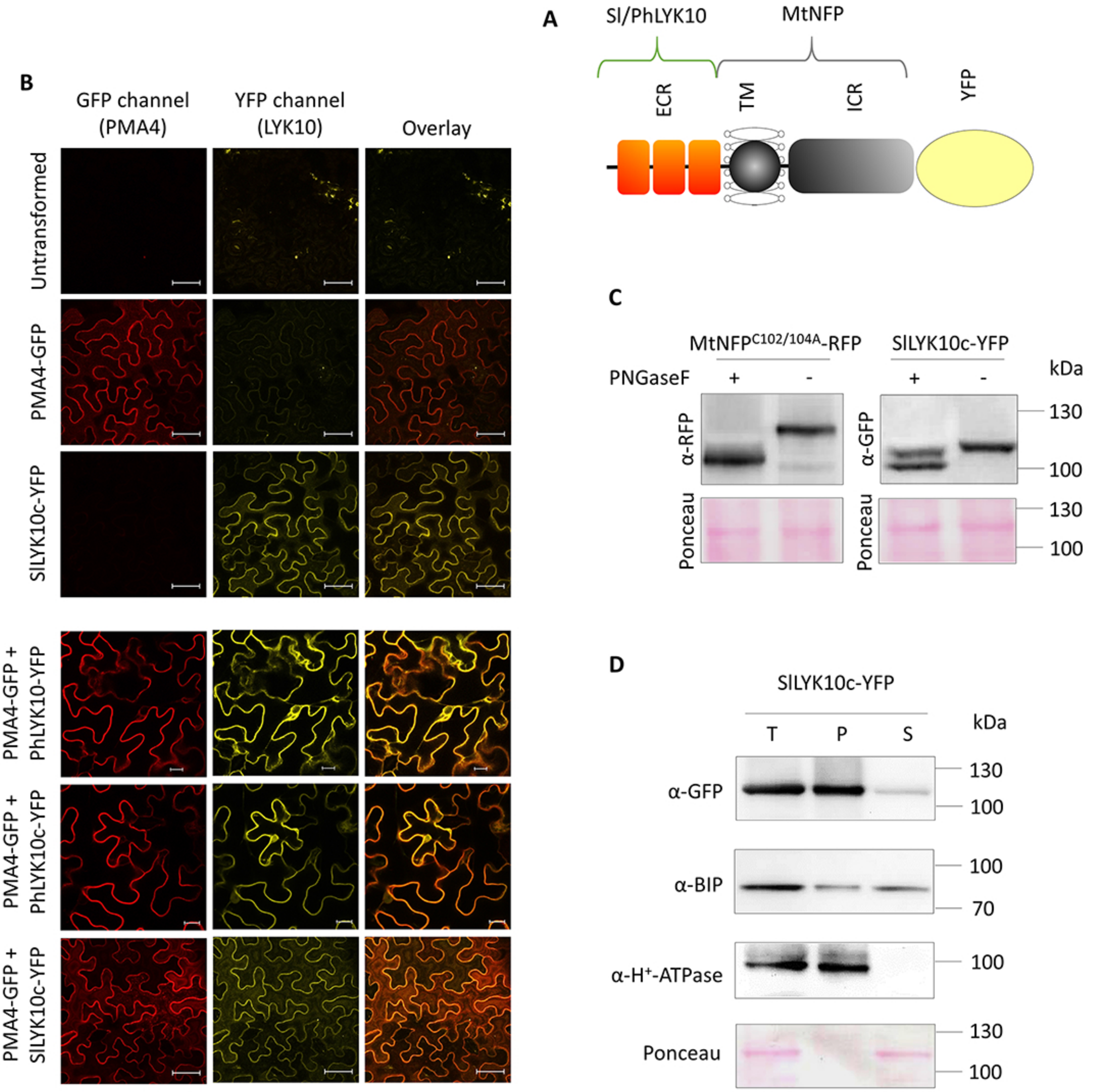

Figure S4 Subcellular localization of SlLYK10c-YFP in *N. benthamiana* leaves. Related to Figure 3. A) Schematic view of the chimeric LYK10c-YFP constructs: the extracellular region (ECR) composed of 3 Lysin Motifs from SlLYK10 or PhLYK10, the transmembrane domain (TM), the intracellular region (ICR) from MtNFP and the yellow fluorescent protein (YFP). B) Co-localization in *N. benthamiana* leaves of PhLYK10-YFP, PhLYK10c-YFP and SlLYK10c-YFP with a plasma membrane (PM) marker. Confocal images of epidermal cells from *N. benthamiana* leaves expressing combinations of PMA4-GFP, a PM marker with PhLYK10-YFP, PhLYK10-YFPc or SlLYK10c-YFP. Fluorescence detected in the GFP and YFP channels is shown in red and yellow respectively, to facilitate visualization of the overlay. Bars represent 20 µm. C) Sensitivity assay of SlLYK10c to PNGaseF. After been processed in the Golgi apparatus, protein N-glycans become insensitive to digestion by PNGaseF. Proteins extracted from *N. benthamiana* leaves were incubated (+) or not (-) with PNGaseF. A higher band after incubation indicates that protein N-glycans are insensitive to the PNGaseF and shows that protein has left the ER. A lower band after incubation indicates that protein N-glycans are sensitive to the PNGaseF digestion and shows that protein did not exit the ER. The mutated form of MtNFP lacking disulfide bridges is retained in the ER [S1] and completely digested by PNGaseF. D) Cell fractionation of leaves expressing SlLYK10c-YFP. Proteins were immunodetected in the total extract (T), the 100,000*g* pellet (P) and the supernatant (S) using anti-GFP antibodies (to detect the YFP fusions), anti-BIP antibodies (to detect an ER marker) or anti-H^+-ATPase antibodies (to detect a PM marker). The Ponceau staining shows that equal amounts of proteins were loaded on the gel.

A

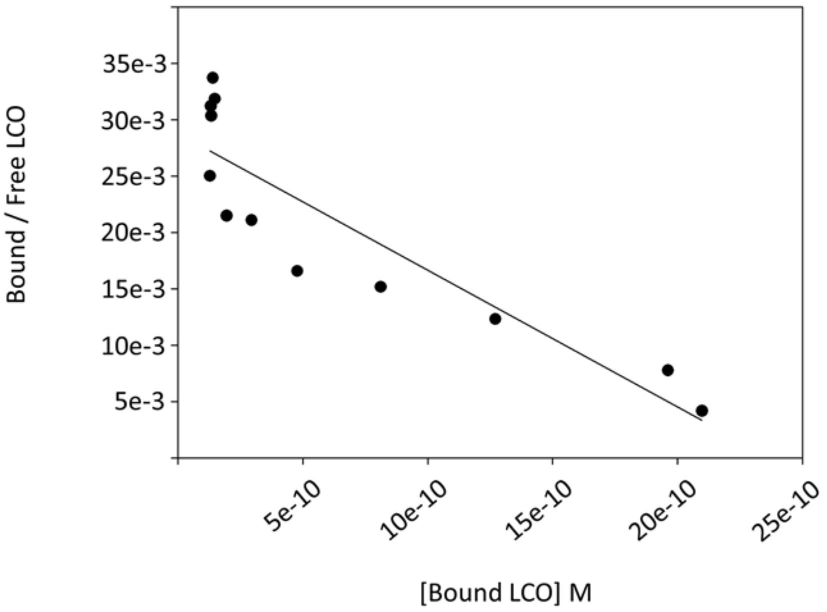

B

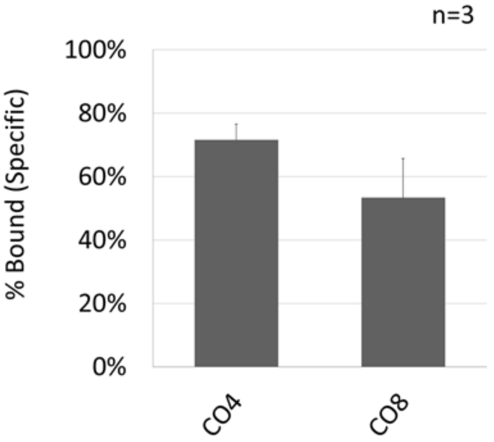

Figure S5 Affinity for LCOs and selectivity for LCOs versus COs of PhLYK10c. **Related to Figure 3.** A) Scatchard plot analysis of a cold saturation experiment using LCO-V(C18:1,NMe,S) and a membrane fraction containing PhLYK10c-YFP. B) Selectivity of the PhLYK10c LCO binding site for various CO structures. Membrane fraction containing PhLYK10c-YFP was incubated with radiolabeled LCO-V(C18:1,NMe,^{35}S) in the presence of 1 µM of the indicated unlabeled COs used as competitors. Non-specific binding was determined in the presence of 1 µM LCO-V(C18:1,NMe,S). Bars represent the percentage of specific binding (means and standard deviations) obtained with independent batches of membrane fractions.

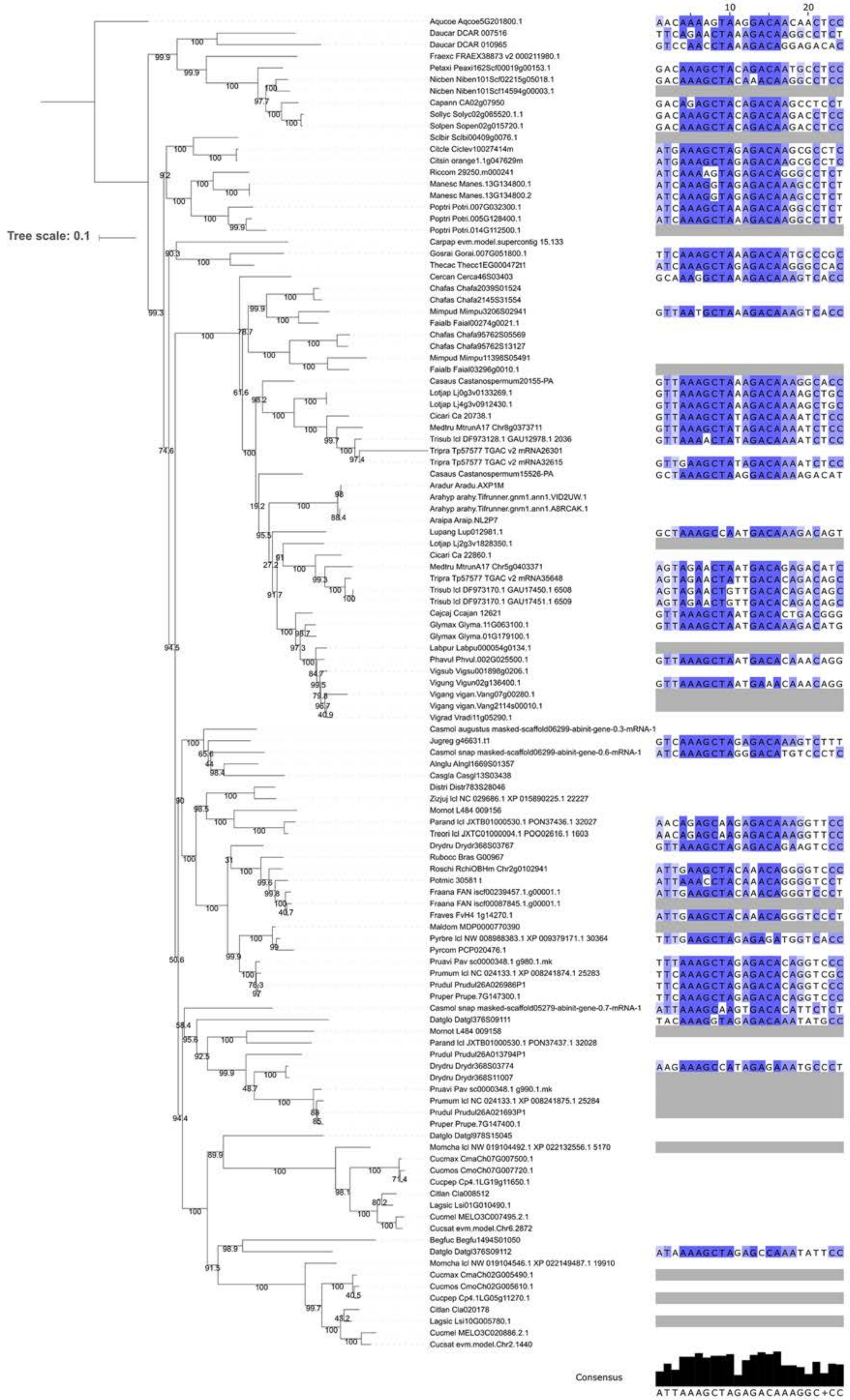

Figure S6 Alignment of the putative *cis*-regulating element in *LYRIA* promoters of 71 dicotyledonous species. **Related to Figure 5.** The tree has been rooted on the basal dicots *Aquilegia coerulea*. Branch support (10,000 replicates of SH-aLRT) is indicated on each branch. Alignment of the putative *cis*-regulating element has been mapped at the right of the tree. Identity percentage is represented by the blue gradient background at each position. Grey and white rectangles indicates that no promoter sequences were extracted for these genes or absence of the motif respectively. Details about the 71 species are given in Table S2.

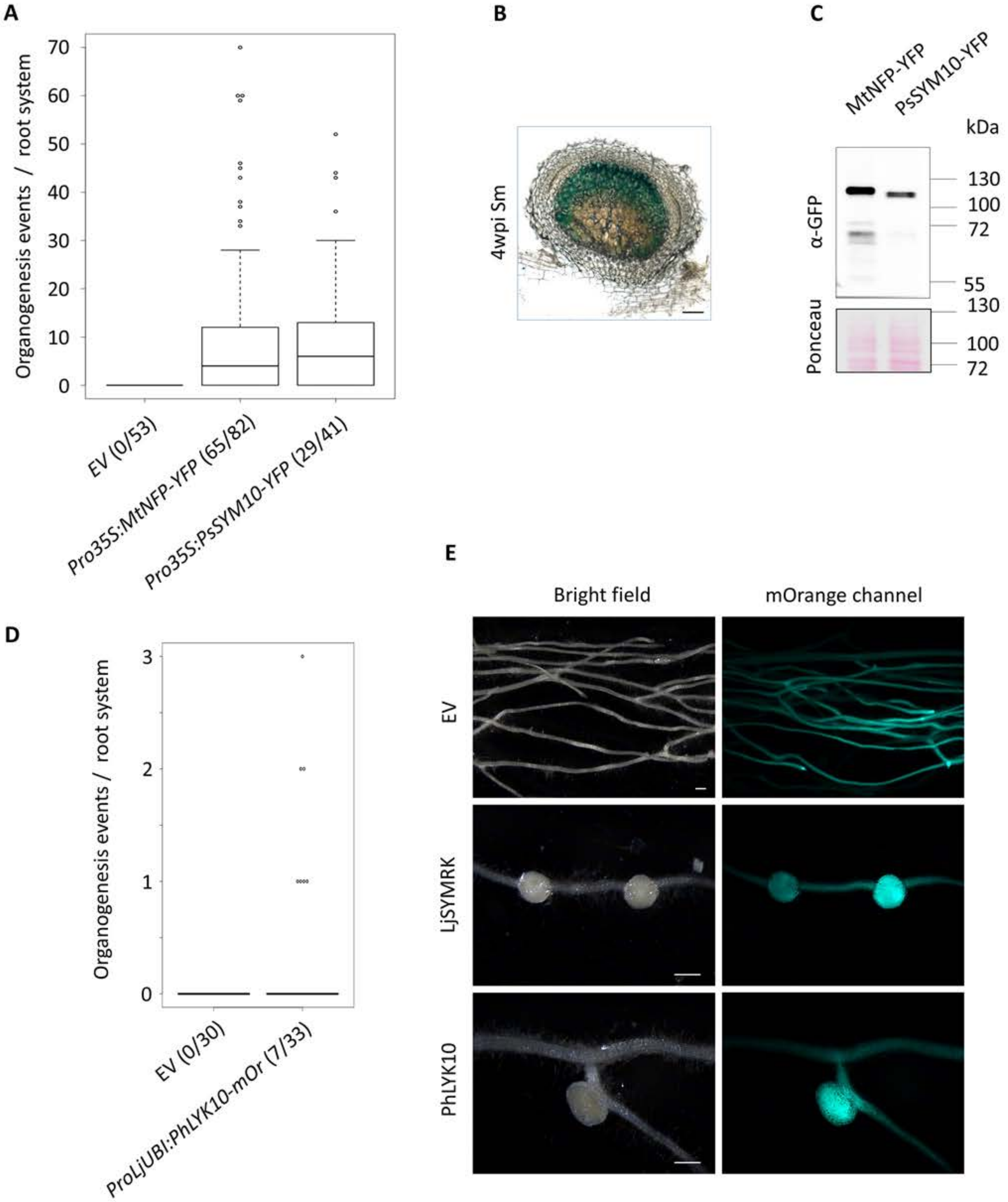

Figure S7 Complementation of *Mtnfp* nodulation by *Pro35S:PsSYM10-YFP* and spontaneous nodulation in *L. japonicus* roots expressing *ProLjUbi:PhLYK10-Orange*. Related to Figure 6. A) Number of organogenesis events (nodules and nodule primordia) 28 dpi with *S. meliloti* lacZ in *Mtnfp* roots transformed with the indicated constructs. Numbers in brackets indicate the numbers of root systems carrying organogenesis events / root systems analyzed. Box plots represent the distribution between individuals from at least 2 independent experiments. Data for the empty vector (EV) and *Pro35:MtNFP-YFP* are the same as in Figure 6A. B) Section of a nodule from roots expressing PsSYM10. *S. meliloti* LacZ were stained by X-Gal. Scale bar represents 100 µm. C) Immunodetection in 20 mg of roots (10 root systems were pooled) expressing the indicated YFP-fusion proteins. D) Roots of *L. japonicus* Gifu wild-type were transformed with the indicated constructs. Box plots represent the number of spontaneous organogenesis events. E) Pictures of spontaneous nodules on *L. japonicus* Gifu roots transformed with EV, *ProLjUBI:LjSYMRK-mOrange* or *ProLjUbi:PhLYK10-mOrange*, 60 days post transformation. Scale bars represent 1 mm.

Subfamily	*Petunia axillaris*	Short name	*Petunia inflata*	Short name	tomato ortholog
**LYR**	Peaxi162Scf00019g00153	PaxLYK10	Peinf101Scf00925g10002	PinLYK10	SlLYK10
	Peaxi162Scf01416g00014	PaxLYK9	Peinf101Scf00428g04012	PinLYK9	SlLYK9
	x		x		SlLYK8
	Peaxi162Scf00420g00445	PaxLYK2	Peinf101Scf01192g06044	PinLYK2	SlLYK2
	Peaxi162Scf00948g00026	PaxLYK15	Peinf101Scf03917g00010	PinLYK15	SlLYK15
	Peaxi162Scf00535g00125*	PaxLYK4	Peinf101Scf00359g10025*	PinLYK4	SlLYK4
	Peaxi162Scf00535g00125*	PaxLYK7	Peinf101Scf00359g10025*	PinLYK7	SlLYK7
	Peaxi162Scf00064g00036	PaxLYK6	Peinf101Scf00471g16004	PinLYK6	SlLYK6
**LYK**	Peaxi162Scf00921g00021	PaxLYK1	Peinf101Scf01180g09048*	PinLYK1A	SlLYK1
	x		Peinf101Scf01180g09048*	PinLYK1B	SlLYK1
	x		x		SlLYK13
	Peaxi162Scf00178g00310*	PaxLYK12	Peinf101Scf00005g00016*	PinLYK12	SlLYK12
	Peaxi162Scf00178g00310*	PaxLYK11	Peinf101Scf00005g00016*	PinLYK11	SlLYK11
	Peaxi162Scf00013g00053	PaxLYK14	Peinf101Scf00500g06038	PinLYK14	SlLYK14
	Peaxi162Scf00038g01033	PaxLYK3	Peinf101Scf02312g02018	PinLYK3	SlLYK3

Table S1 LysM-RLKs in *Petunia axillaris* and *Petunia inflata* genomes. Related to Figure 2.
* Genes in tandem for which the gene model predicts only one gene.

Species	Order	Family	Abbreviation	Reference	RNS	AM
Alnus glutinosa	Fagales	Betulaceae	Alnglu	10.1126/science.aat1743	1	1
Aquilegia coerulea	Ranunculales	Ranunculaceae	Aqucoe	10.7554/eLife.36426	0	ND
Arachis duranensis	Fabales	Fabaceae	Aradur	10.1038/ng.3517	1	1
Arachis hypogaea	Fabales	Fabaceae	Arahyp	peanutbase.org	1	1
Arachis ipaensis	Fabales	Fabaceae	Araipa	10.1038/ng.3517	1	1
Begonia fuchsioides	Cucurbitales	Begoniaceae	Begfuc	10.1126/science.aat1743	0	1
Cajanus cajan	Fabales	Fabaceae	Cajcaj	10.1038/nbt.2022.	1	1
Capsicum annuum cvCM334	Solanales	Solanaceae	Capann	10.1038/ng.2877	0	1
Carica papaya	Brassicales	Caricaceae	Carpap	10.1038/nature06856	0	1
Castanea mollissima	Fagales	Fagaceae	Casmol	www.hardwoodgenomics.org	0	1
Castanospermum australe	Fabales	Fabaceae	Casaus	10.1126/science.aat1743	0	1
Casuarina glauca	Fagales	Casuarinaceae	Casgla	10.1126/science.aat1743	1	1
Cercis canadensis	Fabales	Caesalpiniaceae	Cercan	10.1126/science.aat1743	0	1
Chamaecrista fasciculata	Fabales	Fabaceae	Chafas	10.1126/science.aat1743	1	1
Cicer arietinum ICC4958	Fabales	Fabaceae	Cicari	10.1038/srep12806	1	1
Citrullus lanatus subsp. vulgaris 97103	Cucurbitales	Cucurbitaceae	Citlan	10.1038/ng.2470	0	1
Citrus clementina	Sapindales	Rutaceae	Citcle	10.1038/nbt.2906	0	1
Citrus sinensis	Sapindales	Rutaceae	Citsin	10.1038/nbt.2906	0	1
Cucumis melo	Cucurbitales	Cucurbitaceae	Cucmel	10.1073/pnas.1205415109	0	1
Cucumis sativus PI183967	Cucurbitales	Cucurbitaceae	Cucsat	10.1038/ng.2801	0	1
Cucurbita maxima	Cucurbitales	Cucurbitaceae	Cucmax	10.1016/j.molp.2017.09.003	0	1
Cucurbita moschata	Cucurbitales	Cucurbitaceae	Cucmos	10.1016/j.molp.2017.09.004	0	1
Cucurbita pepo	Cucurbitales	Cucurbitaceae	Cucpep	http://cucurbitgenomics.org	0	1
Datisca glomerata	Cucurbitales	Dasticaceae	Datglo	10.1126/science.aat1743	1	1
Daucus carota	Apiales	Apiaceae	Daucar	10.1038/ng.3565	0	1
Discaria trinervis	Rosales	Rhamnaceae	Distri	10.1126/science.aat1743	1	1
Dryas drummondii	Rosales	Rosaceae	Drydru	10.1126/science.aat1743	1	1
Faidherbia albida	Fabales	Fabaceae	Faialb	10.1093/gigascience/giy152	1	1
Fragaria vesca	Rosales	Rosaceae	Fraves	10.1093/gigascience/gix124	0	1
Fragaria x ananassa	Rosales	Rosaceae	Fraana	www.rosaceae.org	0	1
Fraxinus excelsior	Lamiales	Oleaceae	Fraexc	10.1038/nature20786	0	1
Glycine max	Fabales	Fabaceae	Glymax	10.1038/nature08670	1	1
Gossypium raimondii	Malvales	Malvaceae	Gosrai	10.1038/nature11798	0	1
Juglans regia	Fagales	Juglandaceae	Jugreg	10.1111/tpj.13207	0	1
Lablab purpureus	Fabales	Fabaceae	Labpur	10.1093/gigascience/giy152	1	1
Lagenaria siceraria	Cucurbitales	Cucurbitaceae	Lagsic	10.1111/tpj.13722	0	1
Lotus japonicus	Fabales	Fabaceae	Lotjap	10.1093/dnares/dsn008	1	1
Lupinus angustifolius	Fabales	Fabaceae	Lupang	10.1111/pbi.12615	1	0
Malus domestica	Rosales	Rosaceae	Maldom	www.rosaceae.org	0	1
Manihot esculenta	Malpighiales	Euphorbiaceae	Manesc	10.1038/nbt.3535	0	1
Medicago truncatula	Fabales	Fabaceae	Medtru	10.1038/s41477-018-0286-7	1	1
Mimosa pudica	Fabales	Fabaceae	Mimpud	10.1126/science.aat1743	1	1
Momordica charantia	Cucurbitales	Cucurbitaceae	Momcha	NCBI	0	1
Morus notabilis	Rosales	Moraceae	Mornot	10.1038/ncomms3445	0	1
Nicotiana benthamiana	Solanales	Solanaceae	Nicben	10.1094/MPMI-06-12-0148-TA	0	1
Parasponia andersonii	Rosales	Cannabaceae	Parand	10.1073/pnas.1721395115	1	1
Petunia axillaris	Solanales	Solanaceae	Petaxi	10.1038/nplants.2016.74	0	1
Phaseolus vulgaris	Fabales	Fabaceae	Phavul	10.1038/ng.3008	1	1
Populus trichocarpa	Malpighiales	Salicaceae	Poptri	10.1126/science.1128691	0	1
Potentilla micrantha	Rosales	Rosaceae	Potmic	10.1093/gigascience/giy010	0	1
Prunus avium	Rosales	Rosaceae	Pruavi	10.1093/dnares/dsx020	0	1
Prunus dulcis	Rosales	Rosaceae	Prudul	www.rosaceae.org	0	1
Prunus mume	Rosales	Rosaceae	Prumum	NCBI	0	1
Prunus persica	Rosales	Rosaceae	Pruper	10.1038/ng.2586	0	1
Pyrus communis	Rosales	Rosaceae	Pyrcom	10.1371/journal.pone.0092644	0	1
Pyrus x bretschneideri	Rosales	Rosaceae	Pyrbre	NCBI	0	1
Ricinus communis	Malpighiales	Euphorbiaceae	Riccom	10.1038/nbt.1674	0	1
Rosa chinensis	Rosales	Rosaceae	Roschi	10.1038/s41588-018-0110-3	0	1
Rubus occidentalis	Rosales	Rosaceae	Rubocc	10.1111/tpj.13215	0	1
Sclerocarya birrea	Sapindales	Anacardiaceae	Sclbir	10.1093/gigascience/giy152	0	1
Solanum lycopersicum	Solanales	Solanaceae	Sollyc	10.1038/nature11119	0	1
Solanum pennellii	Solanales	Solanaceae	Solpen	10.1038/ng.3046	0	1
Theobroma cacao	Malvales	Malvaceae	Thecac	10.1186/gb-2013-14-6-r53	0	1
Trema orientalis	Rosales	Cannabaceae	Treori	10.1073/pnas.1721395115	0	1
Trifolium pratense	Fabales	Fabaceae	Tripra	10.1038/srep17394	1	1
Trifolium subterraneum	Fabales	Fabaceae	Trisub	NCBI	1	1
Vigna angularis	Fabales	Fabaceae	Vigang	10.1038/srep080669	1	1
Vigna radiata	Fabales	Fabaceae	Vigrad	10.1038/ncomms6443	1	1
Vigna subterranea	Fabales	Fabaceae	Vigsub	10.1093/gigascience/giy152	1	1
Vigna unguiculata	Fabales	Fabaceae	Vigung	Phytozome	1	1
Ziziphus jujuba cv. Dongzao	Rosales	Rhamnaceae	Zizjuj	10.1038/ncomms6315	0	1

Table S2 List of investigated species for promoter analysis. Related to Figure 5.

Supplemental reference

S1: Lefebvre, B., Klaus-Heisen, D., Pietraszewska-Bogiel, A., Herve, C., Camut, S., Auriac, M.-C., Gasciolli, V., Nurisso, A., Gadella, T.W.J., and Cullimore, J. (2012). Role of N-Glycosylation Sites and CXC Motifs in Trafficking of Medicago truncatula Nod Factor Perception Protein to Plasma Membrane. J Biol Chem *287*, 10812-10823.
