# Supplementary material for: LCO Receptors Involved in Arbuscular Mycorrhiza Are Functional for Rhizobia Perception in Legumes
Source: Curr Biol. 2019 Dec 16;29(24):4249–4259.e5. doi: 10.1016/j.cub.2019.11.038 (PMC6926482; doi:10.1016/j.cub.2019.11.038)
Supplement: Document S2. Article plus Supplemental Information [file mmc2.pdf]

Article

Current Biology

LCO Receptors Involved in Arbuscular Mycorrhiza Are Functional for Rhizobia Perception in Legumes

Highlights

- Mutants in Solanaceaous LysM receptors *LYK10* are impaired in arbuscular mycorrhiza
- LYK10 proteins have a high affinity for lipo-chitooligosaccharidic signal molecules
- *LYK10* promoter is expressed in arbuscule-containing cells in tomato roots
- Solanaceaous *LYK10* can restore nodulation in legumes mutated in their orthologs

Authors

Ariane Girardin, Tongming Wang, Yi Ding, ..., Pierre-Marc Delaux, Jean-Jacques Bono, Benoit Lefebvre

Correspondence

benoit.lefebvre@inra.fr

In Brief

Soil rhizobial bacteria and arbuscular mycorrhizal (AM) fungi produce lipo-chitooligosaccharidic (LCO) signal molecules. Girardin et al. show that plant LCO receptors are involved in establishment of the ancient AM symbiosis and have been recruited during evolution for establishment of the nitrogen-fixing root nodule symbiosis with rhizobia.

Girardin et al., 2019, Current Biology *29*, 4249–4259
December 16, 2019 © 2019 The Author(s). Published by Elsevier Ltd.
https://doi.org/10.1016/j.cub.2019.11.038

CellPress

Current Biology
Article

LCO Receptors Involved in Arbuscular Mycorrhiza Are Functional for Rhizobia Perception in Legumes

Ariane Girardin,[1,8] Tongming Wang,[1,8,9] Yi Ding,[1] Jean Keller,[2] Luis Buendia,[1] Mégane Gaston,[1] Camille Ribeyre,[1] Virginie Gasciolli,[1] Marie-Christine Auriac,[1,3] Tatiana Vernié,[2] Abdelhafid Bendahmane,[4] Martina Katharina Ried,[5,10] Martin Parniske,[5] Patrice Morel,[6] Michiel Vandenbussche,[6] Martine Schorderet,[7] Didier Reinhardt,[7] Pierre-Marc Delaux,[2] Jean-Jacques Bono,[1] and Benoit Lefebvre[1,11,*]
[1]LIPM, Université de Toulouse, INRA, CNRS, 31326 Castanet-Tolosan, France
[2]Laboratoire de Recherche en Sciences Végétales, Université de Toulouse, CNRS, UPS, Auzeville, BP42617, 31326 Castanet-Tolosan, France
[3]Institut Fédératif de Recherche 3450, Université de Toulouse, CNRS, UPS, Plateforme Imagerie TRI-Genotoul, 31326 Castanet-Tolosan, France
[4]IPS2, Institute of Plant Science, INRA, Paris-Saclay, 91190 Gif-sur-Yvette, France
[5]Genetics, Faculty of Biology, University of Munich (LMU), 82152 Martinsried, Germany
[6]Laboratoire Reproduction et Développement des Plantes, Université de Lyon, ENS de Lyon, UCB Lyon 1, CNRS, INRA, F-69342 Lyon, France
[7]Department of Biology, University of Fribourg, 1700 Fribourg, Switzerland
[8]These authors contributed equally
[9]Present address: Rice Research Institute, Key Laboratory of Application and Safety Control of Genetically Modified Crops, Academy of Agricultural Sciences, Southwest University, Chongqing 400715, China
[10]Present address: Structural Plant Biology Laboratory, Department of Botany and Plant Biology, University of Geneva, Geneva, Switzerland
[11]Lead Contact
*Correspondence: benoit.lefebvre@inra.fr
https://doi.org/10.1016/j.cub.2019.11.038

SUMMARY

Bacterial lipo-chitooligosaccharides (LCOs) are key mediators of the nitrogen-fixing root nodule symbiosis (RNS) in legumes. The isolation of LCOs from arbuscular mycorrhizal fungi suggested that LCOs are also signaling molecules in arbuscular mycorrhiza (AM). However, the corresponding plant receptors have remained uncharacterized. Here we show that petunia and tomato mutants in the LysM receptor-like kinases *LYK10* are impaired in AM formation. Petunia and tomato LYK10 proteins have a high affinity for LCOs (*K*d in the nM range) comparable to that previously reported for a legume LCO receptor essential for the RNS. Interestingly, the tomato and petunia *LYK10* promoters, when introduced into a legume, were active in nodules similarly to the promoter of the legume orthologous gene. Moreover, tomato and petunia *LYK10* coding sequences restored nodulation in legumes mutated in their orthologs. This combination of genetic and biochemical data clearly pinpoints Solanaceous LYK10 as part of an ancestral LCO perception system involved in AM establishment, which has been directly recruited during evolution of the RNS in legumes.

INTRODUCTION

Arbuscular mycorrhiza (AM) is an ancient mutualistic symbiosis between Glomeromycota fungi and the majority of land plants, in which fungi provide plants with nutrients acquired from the soil in exchange for carbohydrates and lipids [1, 2]. To colonize plant roots, arbuscular mycorrhizal fungi (AMFs) first cross epidermal and outer cortical cells and then spread inter- or intra-cellularly within roots. Inside inner root cortical cells, AMFs form highly branched structures called arbuscules, across which most nutrient exchange takes place. In the more recent nitrogen-fixing root nodule symbiosis (RNS) that occurs between legumes and rhizobia, the bacteria can fix gaseous nitrogen inside the root nodules. Although the microorganisms are different between these two endosymbioses, the RNS is thought to have evolved through recruitment of genes implicated in the more ancient AM [3].

Nodule organogenesis and bacterial colonization rely on the secretion of lipo-chitooligosaccharide (LCO) signaling molecules by rhizobia [4]. All the rhizobial LCOs have a core structure of 4/5 *N*-acetyl glucosamine (GlcNAc) units of which the terminal non-reducing sugar is substituted with an acyl chain. Additional substitutions, which are important for host specificity, are characteristic of each bacterial strain [5]. Rhizobial LCOs are perceived by Lysin motif receptor-like kinases (LysM-RLKs) that are encoded by a multigenic family, some of which have the ability to bind LCOs [6–8]. Members of the LysM-RLK *LYRIA* phylogenetic group (Figure S1A) [9], such as *Medicago truncatula NFP* (*MtNFP*) or *Lotus japonicus NFR5* (*LjNFR5*), are required for activation of a signaling pathway leading to oscillations of the nuclear Ca^{2+} concentration (Ca^{2+} spiking), nodule organogenesis, and bacterial colonization [10–12].

Two lines of evidence suggest that AM establishment also involves LCO-mediated signaling. The first line is the identification of LCOs from AMFs, and the second is the identification of potential plant LCO receptors. LCOs isolated from AMFs by Maillet

This is an open access article under the CC BY license (http://creativecommons.org/licenses/by/4.0/).

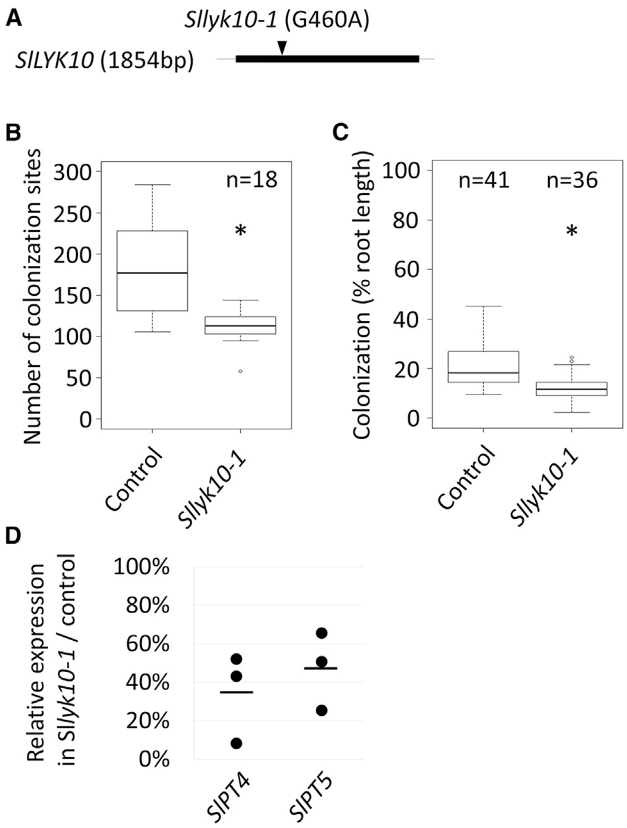

Figure 1. *Sllyk10-1* Is Affected in AMF Colonization

(A) Schematic representation of *SlLYK10*. The thick line represents the single exon. Arrowhead indicates the position of the mutation in *Sllyk10-1*.

(B) Number of AMF colonization sites per root system. Boxplots represent the distribution between individuals from one experiment.

(C) Root-length colonization. Boxplots represent the distribution between root systems from three independent experiments.

(D) Relative expression of the plant AM-marker genes in *Sllyk10-1* versus control roots measured by qRT-PCR. RNAs were extracted from pools of four root systems. The line represents the mean, and the dots represent each replicate.

Statistical differences were calculated using a Kruskal Wallis test in (B) and (C). See also Figures S1 and S2.

et al. (hereafter collectively referred to as Myc-LCOs) have a core structure similar to the rhizobial LCOs and can be sulfated or not on the reducing sugar [13]. Exogenous application of these Myc-LCOs both increases the level of AMF root colonization [13] and activates Ca^{2+} spiking in various plant species [14, 15]. Short-chain chitooligosaccharides (COs) produced by AMFs can also activate Ca^{2+} spiking [16], indicating that both LCOs and short-chain COs have the potential to be involved in partner recognition during AM. However, whether Myc-LCOs and/or short-chain COs are indeed involved in AM establishment is not known.

Several LysM-RLKs (*Parasponia andersonii PanNFP1* and/or *PanNFP2*, tomato *SlLYK10* and *SlLYK12*, *Medicago truncatula MtLYK9*, and rice *OsCERK1*) have been shown to be involved in AM [17–22], but their LCO/CO binding properties have not been determined so far. *SlLYK12, MtLYK9*, and *OsCERK1* belong to the *LYKI* phylogenetic group (Figure S1B [9]). These LysM-RLKs are likely co-receptors, since *MtLYK9* and *OsCERK1* have a dual function in AM and defense [19, 20, 23], and OsCERK1 is involved in perception of various ligands including short-chain COs, chitin, and peptidoglycan [24–26], the latter two being components of fungal and bacterial cell walls, respectively, known as plant defense elicitors. The other LysM-RLKs known to control AM belong to the *LYRIA* group that contains members only in plant species that establish AM and/or RNS [27, 28]. In tomato, virus-induced silencing of the unique *LYRIA* gene (*SlLYK10*) resulted in significantly lower levels of AM colonization [21].

Although the current hypothesis is that the RNS evolved by coopting genes involved in the AM [3], it is unclear how LCO receptors may have evolved to become key players in RNS establishment.

Here, we functionally characterize LCO receptors from Solanaceae, a plant family that establishes AM but not RNS. We use heterologous expression in legumes to infer an evolutionary scenario of LCO receptor recruitment for RNS. Our data suggest that non-legume *LYRIA* genes encode LCO receptors involved in AM and that the transcriptional regulation required for LCO receptor function in RNS has been directly co-opted from AM.

RESULTS

The Petunia and Tomato LYRIA Genes Are Involved in AM Establishment

We have previously shown that knockdown of the *LYRIA* gene in tomato (*SlLYK10*) resulted in impaired AM establishment [21]. Because of the limitations of gene silencing, we screened an EMS-mutagenized tomato population and identified a line carrying a missense mutation in *SlLYK10* affecting the second LysM ($E^{154}K$) (Figure 1A). Segregants of this line with a homozygous mutation (*Sllyk10-1*) displayed reduced numbers of AMF colonization sites, root-length colonization, and expression of AM-marker genes (Figures 1B–1D) compared with segregants with a WT *SlLYK10* allele (control).

We also searched for knockout lines in a related Solanaceae species, *Petunia hybrida*, by screening a transposon-mutagenized population [29]. We identified a line with a *dTPh1* insertion in the *SlLYK10* ortholog *PhLYK10* (Figures 2A and S2), which segregated with the expected 1:2:1 wild-type:heterozygous:homozygous ratio (Figure 2B). Segregants with a homozygous *dTph1* insertion (*Phlyk10-1*) displayed a reduced number of AMF colonization sites (Figure 2C), many of them being impaired in arbuscule formation (Figure 2D), compared with segregants with a WT *PhLYK10* allele (control). Confocal microscopy analysis of colonized cells showed hyphal coils instead of arbuscules (Figure 2E). The ratio of colonization sites with aberrant arbuscule development was significantly higher in *Phlyk10-1* plants (Figure 2F). The *Phlyk10-1* plants also displayed a reduced level of root-length colonization and expression of AM-marker genes (Figures 2G and 2H). Furthermore, in a segregating population, we found that increased numbers of colonization sites with aberrant arbuscule development correlated with the presence of the *dTph1* insertion (Figure 2I). Unexpectedly, heterozygous individuals also showed impaired arbuscule development. This, together with the phenotypic similarity observed in *SlLYK10*-silenced plants [21] and the nature of the mutation (stop codon

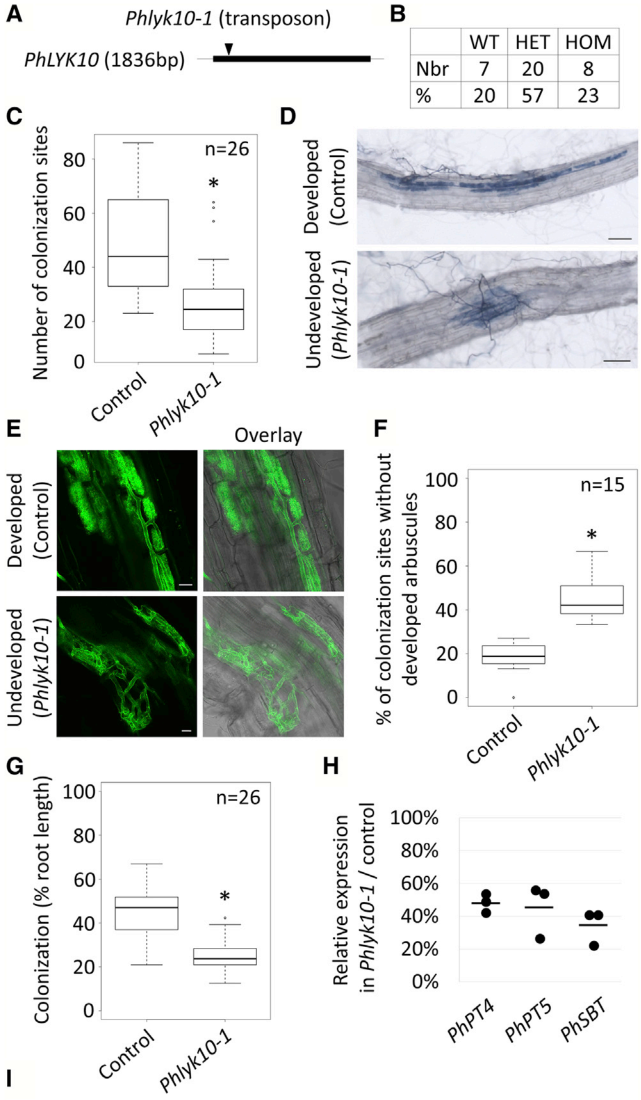

	WT	HET	HOM
Nbr	7	20	8
%	20	57	23

	WT (n=24)	HET (n=10)	HOM (n=12)
% of colonization sites without developed arbuscules	18.3^{b}	43.3^{a}	42.6^{a}
SD	10.5	16.3	14.2

Figure 2. *Phlyk10-1* Is Affected in AMF Colonization and Arbuscule Formation

(A) Schematic representation of *PhLYK10*. The thick line represents the single exon. Arrowhead indicates the position of the *dTph1* insertion in *Phlyk10-1*.

(B) Number of wild-type (WT), heterozygous (HET), and homozygous (HOM) individuals for the *dTph1* insertion on progenies of HET F2 plants after a backcross. No significant difference with theoretical segregation was found.

(C) Number of AMF colonization sites per root system. Boxplots represent the distribution between individuals from three independent experiments.

(D) Images of ink-stained colonization sites.

(E) Images of WGA-CF488A-stained AMF.

(F) Percentage of colonization sites without developed arbuscules (as in D) versus the total number of colonization sites. Boxplots represent the distribution between root systems from one experiment.

(G) Root-length colonization. Boxplots represent the distribution between root systems from one experiment.

(H) Relative expression of the plant AM-marker genes in *Phlyk10-1* versus control roots measured by qRT-PCR. RNAs were extracted from pools of at least three root systems. The line represents the mean, and the dots represent each replicate.

(I) Same as in (F) except that measured on progenies of HET F2 plants after a backcross. Individual plants were genotyped and phenotyped. Means and SDs are shown in the table.

Statistical differences were calculated using a Xhi2 test in (B), a Student's t test in (C), (F), and (G), or a Kruskal Wallis test in (I). Scale bars represent 100 μm in (D) and 20 μm in (E). See also Figures S1 and S2 and Table S1.

in *dTPh1* close to the start codon of *PhLYK10*), suggests that *PhLYK10* function is sensitive to gene dosage.

LCO Binding by LYRIA Proteins Predates the Evolution of RNS

LCO-binding in legume *LYRIA* proteins may have originated from ancestral LCO-binding proteins, or it may have been gained in legumes as a key property in the evolution of the RNS. To discriminate between these two possibilities, we determined the LCO-binding properties of SlLYK10 and PhLYK10. We used *Agrobacterium tumefaciens*-mediated transient expression to produce SlLYK10-YFP and PhLYK10-YFP in leaves of *Nicotiana benthamiana*, a plant protein expression system which allows the formation of disulfide bridges essential for LysM-RLK function [30, 31]. SlLYK10-YFP was localized in undefined cytoplasmic structures in *N. benthamiana* leaf cells, although the protein was properly localized at the plasma membrane (PM) in transgenic tomato roots (Figures S3A–S3D). We previously observed that a chimeric LysM-RLK was well localized at the PM in *N. benthamiana* leaves and had LCO-binding properties similar to the corresponding full-length protein [6]. We thus generated a chimera (hereafter referred to as SlLYK10c) (Figure S4A) composed of SlLYK10 extracellular region (ECR) and MtNFP intracellular region. Although a fraction of SlLYK10c-YFP was localized to the endoplasmic reticulum (ER) of *N. benthamiana* leaf cells (Figure 3A), both co-localization with a PM marker and the analysis of N-glycan maturation indicated that a significant fraction of the proteins reached the PM (Figures S4B–S4D). Subcellular localization of PhLYK10 and a PhLYK10 chimera (PhLYK10c) was similar (Figures 3A and S4B).

SlLYK10c-YFP, PhLYK10-YFP, and PhLYK10c-YFP were all immunodetected in the membrane fractions extracted from *N. benthamiana* leaves (Figure 3B). Their affinity to LCOs was determined by radio-ligand binding assays using LCO-V(C18:1,NMe) labeled with ^{35}S. Specific binding of LCOs to membrane fractions was detected in extracts of leaves expressing *PhLYK10-YFP*, *PhLYK10c-YFP*, or *SlLYK10c-YFP* but not in extracts of untransformed leaves (Figure 3C).

The affinity of PhLYK10-YFP and SlLYK10c-YFP for LCO-V(C18:1,NMe,S) was determined by a cold saturation experiment. Scatchard plot analysis revealed single class of binding sites (Figure 3D) with dissociation constants (K_d) of 22 nM ± 5 nM (n = 3) and 19 nM ± 4 nM (n = 3), for PhLYK10 and SlLYK10c, respectively, showing that both proteins exhibit high-affinity binding to this LCO. Their selectivity toward COs was then determined through competition assays between the ^{35}S-LCO and an excess (1 μM) of unlabeled COs. CO4 and CO8 were much less efficient competitors of ^{35}S-LCO binding (Figure 3E) with inhibitory constants (K_i) higher than 1 μM,

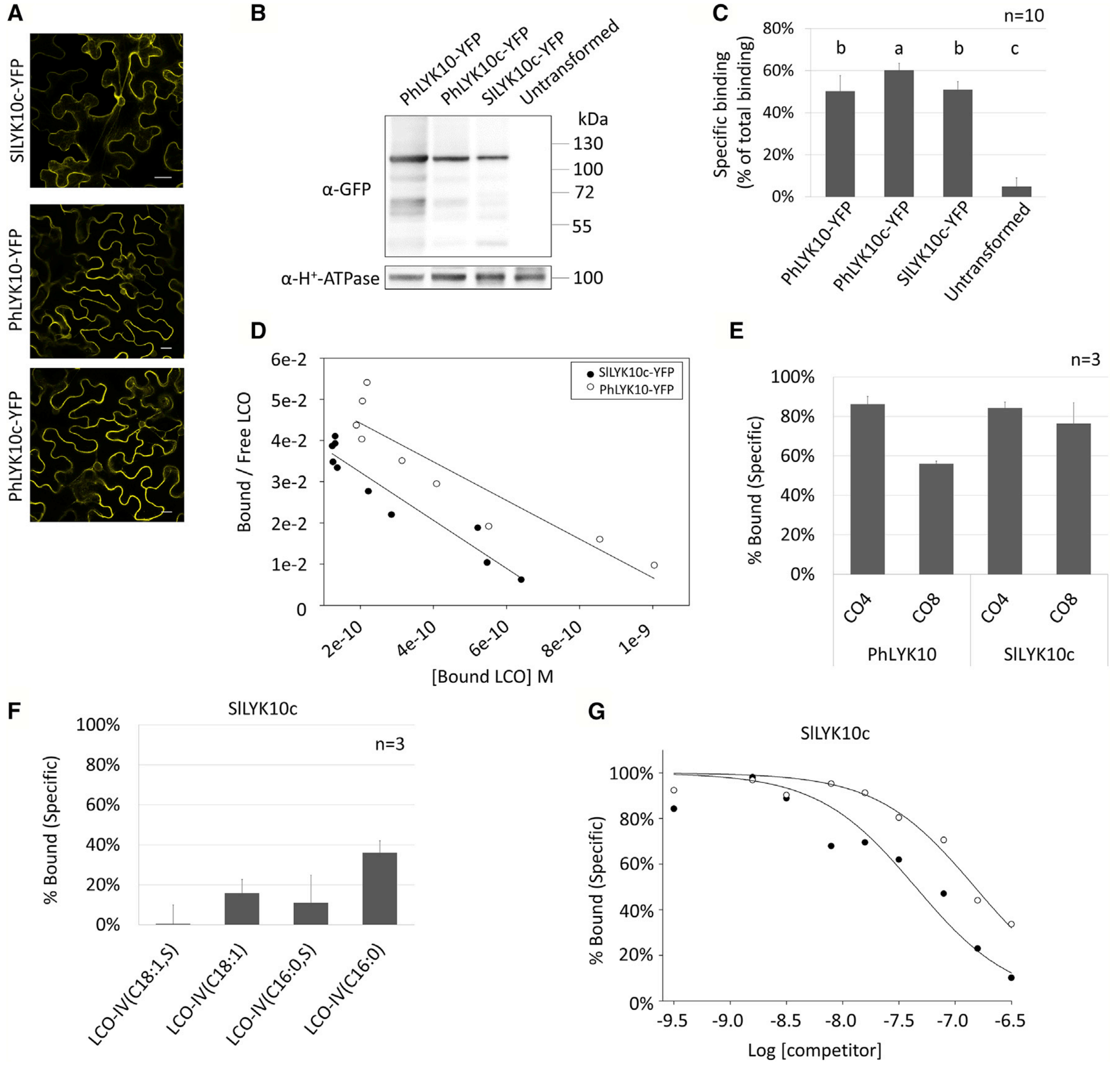

Figure 3. PhLYK10 and SlLYK10c Have a High Affinity for LCOs and Discriminate LCOs versus COs

(A) Confocal images of epidermal cells from *N. benthamiana* leaves expressing the indicated proteins. Scale bars represent 20 μm.

(B) Immunodetection of the YFP-fusion proteins in 10 μg of membrane fractions from *N. benthamiana* leaves.

(C) Binding of LCO-V(C18:1,NMe,^{35}S) to membrane fractions containing the indicated proteins. Incubation with the radiolabeled ligand in the absence or in the presence of 1 μM unlabeled LCO-V(C18:1,NMe,S) allowed to determine the total and non-specific binding respectively and by difference the specific binding. The specific binding is expressed as a percentage of the total binding to normalize variations in protein expression level between biological replicates. Means and standard deviations between replicates are shown.

(D) Scatchard plot analysis of cold saturation experiments using a range of concentration of LCO-V(C18:1,NMe,S) as competitor. The plots are representative of experiments performed with three independent batches of membrane fractions.

(E) Selectivity of the PhLYK10 and SlLYK10c LCO-binding sites for LCOs versus COs. Membrane fractions were incubated with LCO-V(C18:1,NMe,^{35}S) in the presence of 1 μM unlabeled CO4 or CO8 as competitors. Non-specific binding was determined with 1 μM LCO-V(C18:1,NMe,S). Bars represent the percentage of specific binding (means and standard deviations) obtained with independent batches of membrane fractions.

(F) Selectivity of the SlLYK10c LCO-binding sites for various Myc-LCO structures. This is the same as in (B) except that the unlabeled competitors are the indicated LCOs.

(G) Competitive inhibition using a range of concentration of Myc-LCO-IV(C16:0,S) (black circles) or Myc-LCO-IV(C16:0) (white circles).

See also Figures S3, S4, and S5.

showing that the LCO-binding site of PhLYK10 and SlLYK10c exhibits a low affinity for COs. We also determined the binding selectivity of SlLYK10c-YFP toward Myc-LCOs. All Myc-LCOs were able to compete the binding of the ^{35}S-LCO (Figure 3F). The affinities of SlLYK10c-YFP for the sulfated and non-sulfated Myc-LCOs were further determined by competition assays. K_i of 192 nM ± 52 nM (n = 3) and 354 nM ± 60 nM (n = 3) were obtained for LCO-IV(C16:0,S) or LCO-IV(C16:0), respectively (Figure 3G).

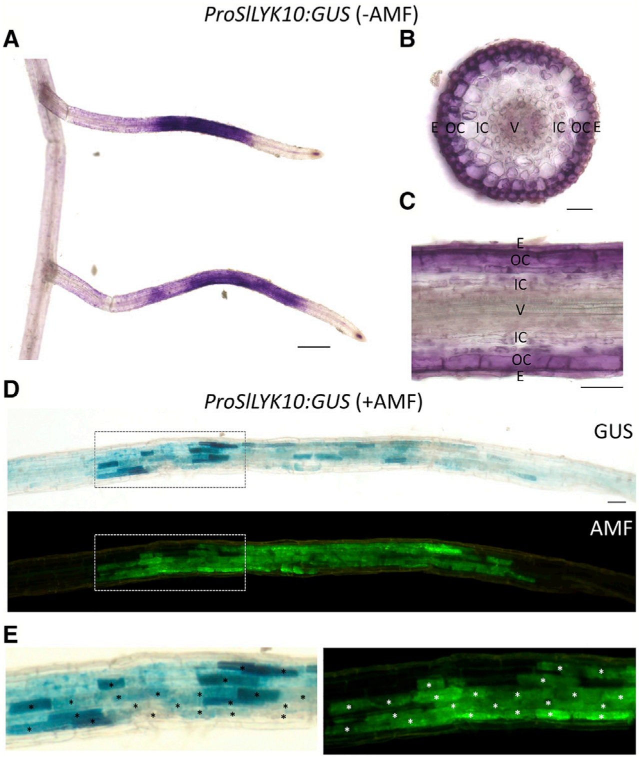

Figure 4. *ProSlLYK10:GUS* Is Expressed in Arbuscule-Containing Cells of Tomato Roots

(A) GUS activity (magenta) in tomato roots from chimeric plants in the absence of AMF.

(B–C) Transversal (B) and longitudinal (C) sections in a root segment showing GUS staining (E, epidermis; OC, outer cortex; IC, inner cortex; V, vessels).

(D) Tomato ROC line colonized by AMF (GUS staining, blue; AMF staining [WGA-CF488A], green).

(E) Close-up of (C). Arbuscule-containing cells are marked by an asterisk.

Scale bars represent 500 μm in (A) and 50 μm in (B–D).

Finally, we found that affinity of PhLYK10c for LCOs (K_d of 60 nM ± 18 nM [n = 2]) and selectivity for LCOs versus COs were similar to that of PhLYK10 (Figures S5A and S5B), confirming that the LCO-binding properties of full-length proteins are conserved in our chimeric LysM-RLK.

Promoters from LYRIA Genes Did Not Neo-functionalize to Support RNS

Evolutionary genetics in various eukaryotic models indicates that recruitment of existing pathways to new traits often involves the gain or loss of *cis*-regulatory elements in promoter regions [32, 33]. We tested whether change in the transcriptional regulation for the *LYRIA* gene occurred for advent of the RNS by analyzing the expression patterns of Solanaceae *LYRIA* promoters in AM and RNS. In un-inoculated transgenic tomato roots, a 1.8 kbp sequence of the *SlLYK10* promoter region (*ProSlLYK10*) drove the expression of the GUS reporter primarily in lateral roots (Figure 4A), the preferred site for AMF penetration [34]. Transverse and longitudinal sections revealed GUS activity in the epidermis and outer cortex (Figures 4B and 4C). In transgenic roots maintained as root organ cultures (ROCs) and inoculated with AMF, GUS staining was observed in arbuscule-containing cells (Figures 4D and 4E). Strongest GUS expression was observed in cells at the border of colonization units. Interestingly, this is the site where young arbuscules develop [35].

During nodulation, the *M. truncatula LYRIA* gene *MtNFP* is expressed in nodule primordia and later in the infection zone of mature nodules [10]. We analyzed the activity of the petunia and tomato *LYRIA* promoters during nodulation in *M. truncatula*. *ProSlLYK10* and *ProPhLYK10* exhibited an activity similar to *ProMtNFP* with GUS staining in the nodule primordia and in the apex of mature nodules (Figure 5A). This shows that the promoters of the two Solanaceae *LYRIA* genes contain all the information required for expression in legume nodules. We also compared the expression patterns of the three promoters in *M. truncatula* mycorrhizal roots. *ProMtNFP* showed a weak non-specific expression, while *ProSlLYK10* and *ProPhLYK10* were mostly active in arbuscule-containing cells (Figure 5A). These results suggest that *ProSlLYK10* and *ProPhLYK10* have the full symbiotic capacity required for expression during AM and RNS and that *ProMtNFP* has lost the ability to drive expression in mycorrhizal roots. In legumes, a whole-genome duplication at the base of the Papilionoideae gave rise to two paralogous *LYRIA* genes in *Medicago*, *MtNFP*, and *MtLYR1*. In contrast to *MtNFP*, *MtLYR1* is expressed in mycorrhizal roots [36], but not in nodules (*M. truncatula* Gene Expression Atlas). The absence of *ProMtNFP* expression in mycorrhizal roots may reflect either a modification of the ancestral gene promoter required for its recruitment for RNS or the sub-functionalization following the gene duplication in the Papilionoideae. To test these possibilities, we analyzed the expression pattern of *MpNFP*, the *LYRIA* gene from *Mimosa pudica*, a legume from the Mimosoideae clade that did not undergo whole genome duplication [37]. We found that in *M. truncatula*, *ProMpNFP* drives a similar expression pattern to *ProSlLYK10* and *ProPhLYK10*, with activity detected both in nodules and in arbuscule-containing cells (Figure 5A). This indicates that the evolution of RNS did not require the loss of *LYRIA* gene activation during AM. To determine whether Solanaceae *LYRIA* promoters are sufficient to provide *LYRIA* protein activity for RNS, we expressed the *MtNFP* coding sequence (CDS) under the control of *ProSlLYK10* in a *Mtnfp* mutant line unable to form nodules. We observed a similar number of nodules in roots containing either the *ProSlLYK10:MtNFP-YFP* construct or the *ProMtNFP:MtNFP-YFP* construct (Figure 5B).

These results suggest that *cis*-regulatory elements essential for expression in nodules are conserved between *ProSlLYK10*, *ProPhLYK10*, *ProMtNFP*, and *ProMpNFP*. To identify the region that contains these *cis*-regulatory elements, we first cloned a shorter version of the *MtNFP* promoter (240 bp before the start codon) and tested its activation during RNS. Similar to the 1.5 kb sequence, this shorter promoter was sufficient to drive expression of the GUS reporter in young nodules (Figure 5C). Through scanning the promoter region of orthologous *LYRIA* genes from nodulating and non-nodulating dicotyledonous species, we identified the AAAGCTANNGACA consensus sequence in the promoters of at least one *LYRIA* gene in 60% of 71 investigated species (Figure S6). This consensus sequence is located in the proximal region of *MtNFP* and *SlLYK10* promoters (Figure 5D). A *SlLYK10* promoter region starting 10 bp upstream of

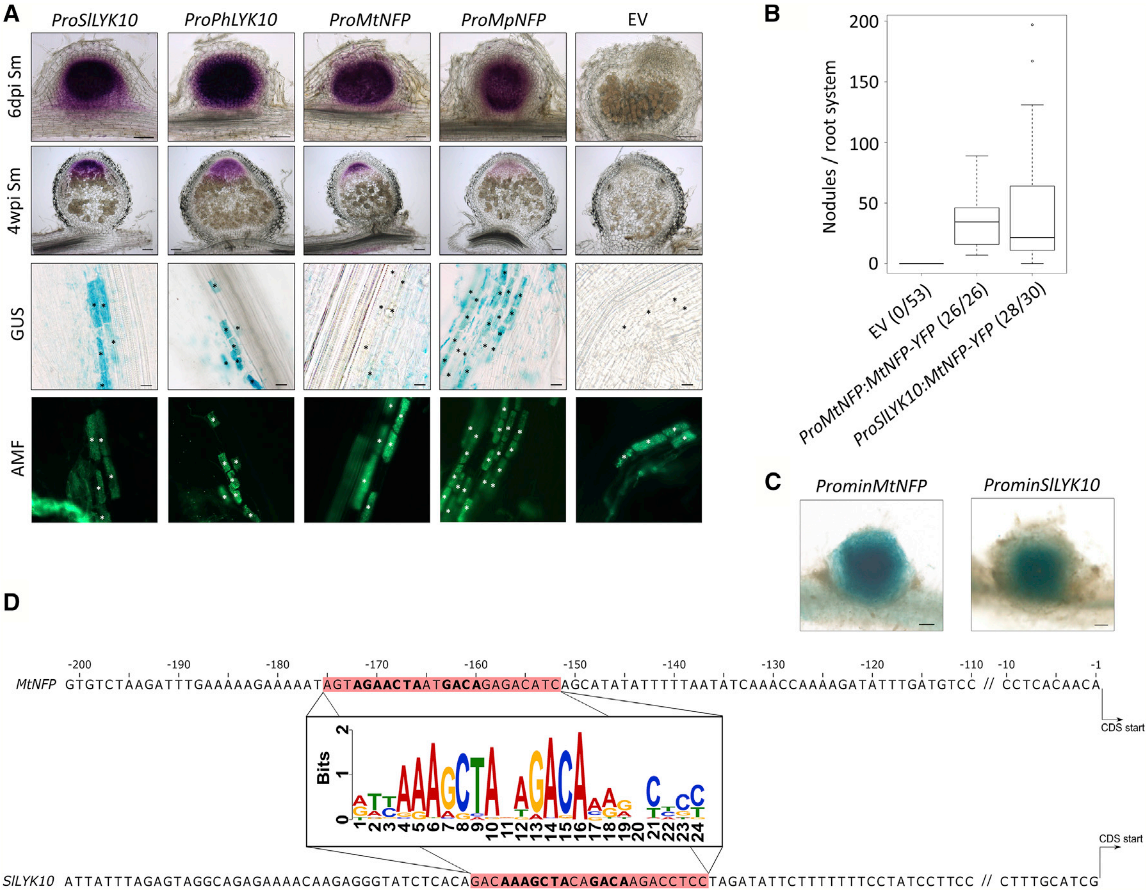

Figure 5. *ProSlLYK10*:GUS and *ProPhLYK10*:GUS Are Expressed in Nodules and in Arbuscule-Containing Cells of *M. truncatula* Roots

(A) GUS staining (magenta) in young and mature nodules of *M. truncatula* transgenic roots containing the indicated constructs or the empty vector (EV) and inoculated with *S. meliloti* (Sm). GUS staining (blue) in arbuscule-containing cells (green) of *M. truncatula* transgenic roots inoculated with *R. irregularis*. Arbuscule-containing cells are marked by an asterisk.

(B) Number of nodules in *Mtnfp* roots complemented by the indicated constructs and inoculated with *S. meliloti*. Numbers in brackets indicate the numbers of root systems carrying nodules/root systems analyzed. Boxplots represent the distribution between individuals from at least two independent experiments. Scale bars represent 100 μm in the nodule sections and 20 μm the right panels.

(C) The GUS reporter (blue) under the control of a minimal *MtNFP* (*ProminNFP*, 240 bp before the start codon) or *SlLYK10* (*ProminSlLYK10*, 185 bp before the start codon) promoters is expressed in young nodules of *M. truncatula* roots inoculated with *S. meliloti*. Scale bars represent 100 μm.

D) The putative *cis*-regulating element in *MtNFP* and *SlLYK10* promoters is highlighted in red in the 200 bp sequences before the start codons. The most conserved positions are in bold. The logo shows the degree of conservation of the putative *cis*-regulating element among 71 dicotyledonous *LYRIA* genes.
See also Figure S6 and Table S2.

this consensus sequence (185 bp before the start codon) also exhibited activity in young nodules (Figure 5C).

Taken together, our results indicate that the recruitment of *LYRIA* genes for RNS did not require modification in the regulation of their expression.

PhLYK10 Partially Complements the Lack of Nodules in Legume Mutants

Besides modifications in *cis*-regulatory elements, recruitment of a gene into a new trait may result from neo-functionalization of the encoded protein [32, 38]. To test whether the recruitment of *LYRIA* genes for RNS involved neofunctionalization, we performed complementation assays of *Mtnfp* and *Ljnfr5* mutants with the CDS of *PhLYK10* and *SlLYK10*. *ProLjNFR5*:*SlLYK10* did not restore nodulation in *Ljnfr5* mutant. This is similar to what was observed in *Mtnfp* mutant with the CDS of *MtNFP* ortholog in pea, *PsSYM10*, under the control of *ProMtNFP* [39]. However, we found that *PsSYM10* under the control of the strong 35S promoter was able to complement *Mtnfp* for nodule formation and rhizobial colonization (Figures S7A and S7B). Strikingly, *Pro35S:SlLYK10* and *Pro35S:PhLYK10* were also able to restore the formation of nodules in *Mtnfp* (Figure 6A) while *Mtnfp* roots expressing AtCERK1, an *A. thaliana* LysM-RLK from the *LYKI* group (Figure S1B) did not produce any nodules. The nodules formed in roots expressing SlLYK10 were fully colonized by rhizobia, similarly to roots expressing MtNFP, while only a very weak rhizobial staining was observed in roots expressing PhLYK10 (Figure 6B). Immunodetection of proteins in *Mtnfp* roots revealed that MtNFP was expressed at the highest level (Figures 6C and S7C), whereas PhLYK10 was below the

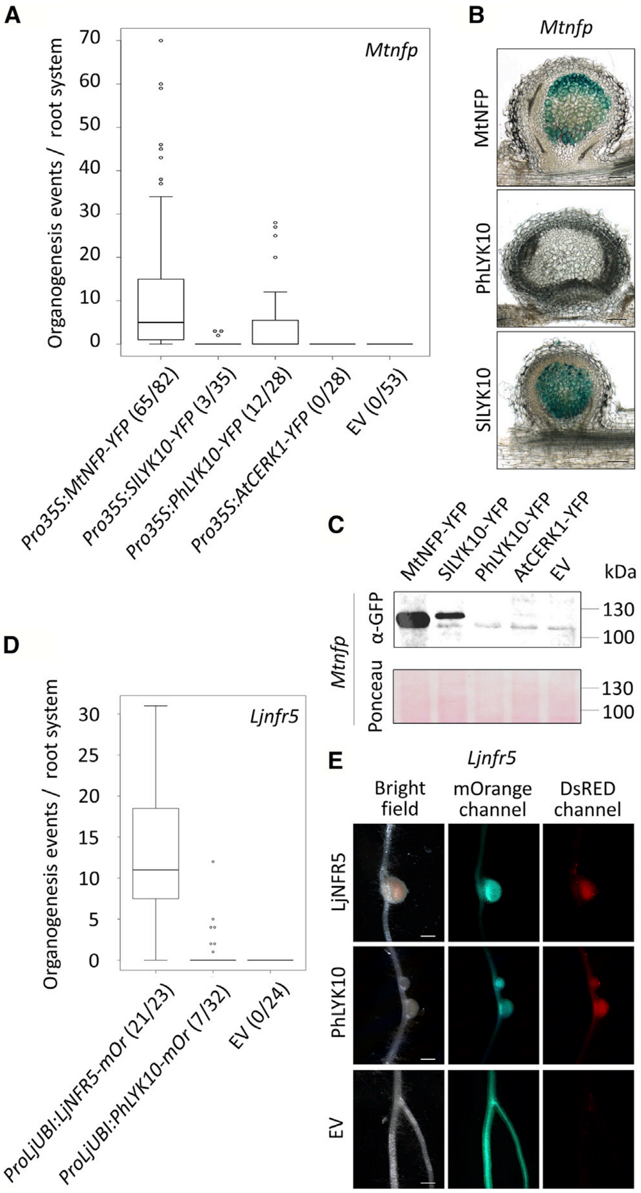

Figure 6. *PhLYK10* Coding Sequence Complements the Lack of Nodulation in *Mtnfp* and *Ljnfr5*

(A) Number of organogenesis events (nodules and nodule primordia) 28 days post inoculation (dpi) with *S. meliloti* lacZ in *Mtnfp* roots complemented by the indicated constructs. Numbers in brackets indicate the numbers of root systems carrying organogenesis events/root systems analyzed. Boxplots represent the distribution among individuals from at least two independent experiments. Data for empty vector (EV) are the same as in Figure 5B.

(B) Sections of nodules from *Mtnfp* roots as in (A). *S. meliloti* LacZ were stained by X-Gal.

(C) Immunodetection of the YFP-fusion proteins in 20 mg of *Mtnfp* roots.

(D) Number of organogenesis events 26 dpi with *M. loti* DsRED in *Ljnfr5* roots complemented by the indicated constructs. Numbers in brackets indicate the numbers of root systems carrying organogenesis events/root systems analyzed.

(E) Images of *Ljnfr5* roots as in (D).

Scale bars represent 100 μm in (B) and 1 mm in (E). See also Figure S7.

detection limit despite its ability to partially complement nodulation in *Mtnfp*. This may reflect differences in the stability of the orthologs in *M. truncatula*, which in turn may explain the different levels of complementation by the different *LYRIA* proteins. Nodulation was also restored in *Ljnfr5* roots expressing PhLYK10 (Figure 6D), although, as in *Mtnfp* roots, fewer nodules were formed compared with complementation with the endogenous *LYRIA* gene. In this case, the nodules were fully colonized by rhizobia (Figure 6E). *ProLjUBI:PhLYK10-mOrange* also triggered spontaneous nodule formation in *L. japonicus* in the absence of rhizobia (Figures S7D and S7E) like overexpression of *LjNFR5* [40].

DISCUSSION

Myc-LCOs can induce gene transcription, Ca^{2+} spiking, and root branching [13–15, 41, 42]. However, until now it was not clear whether they are involved in AM establishment. Here, we demonstrate high-affinity LCO-binding properties of PhLYK10 and SlLYK10, which, together with the mycorrhizal phenotype of the *Phlyk10-1* and *Sllyk10-1* mutant lines, provide the strongest evidence to date that Myc-LCOs are directly involved in AM establishment.

Detailed characterization of PhLYK10 and SlLYK10 revealed that they are high-affinity LCO-binding proteins that discriminate LCOs versus COs; their affinity for LCOs being as high as that of the previously characterized legume *LYRIA* protein, LjNFR5, expressed in the same heterologous system [8]. SlLYK10 recognized the Myc-LCO structures described in [13] with similar affinity for sulfated and non-sulfated Myc-LCOs. However, SlLYK10 exhibited a higher affinity for LCO-V(C18:1,NMe,S) compared with the published Myc-LCO structures, indicating that such LCOs or related structures could potentially represent additional Myc-LCOs.

The similarity of the AM phenotype in the petunia line knockout for *PhLYK10*, the tomato line bearing a point mutation in *SlLYK10*, and the tomato *SlLYK10*-silenced plants [21] provides compelling evidence that the *LYRIA* gene is involved in AM establishment in Solanaceae. Reduction in the number of colonization sites in the above-mentioned plants suggests a role at early stages for AMF penetration in roots. Moreover, the aberrant arbuscule development observed in *Phlyk10-1* and *SlLYK10*-silenced plants suggests an additional role in arbuscule development. The activity of the *SlLYK10* promoter in tomato roots initially in the epidermis and upon colonization in arbuscule-containing cells further supports a role of the *LYRIA* gene at several steps of AM establishment in Solanaceae.

Although *Phlyk10-1*, *Sllyk10-1*, and the *SlLYK10*-silenced plants are affected in AM establishment, AMFs can still colonize roots and form arbuscules. In a mutant of the rice *LYRIA* gene *OsNFR5*, AM-marker gene expression was decreased, but the number of AMF colonization sites was not affected [18]. Mutants in *MtNFP* are also colonized normally by AMFs [19, 43] despite an almost complete block of symbiosis-related responses to both rhizobial LCOs and Myc-LCOs [13, 14, 43, 44]. Moreover, a double mutant in the two *LYRIA* genes *LjNFR5* and *LjLYS11* was not affected in AM establishment [45]. Altogether, this suggests redundancy at the level of LCO perception or that other signals could activate the LCO-mediated signaling pathway.

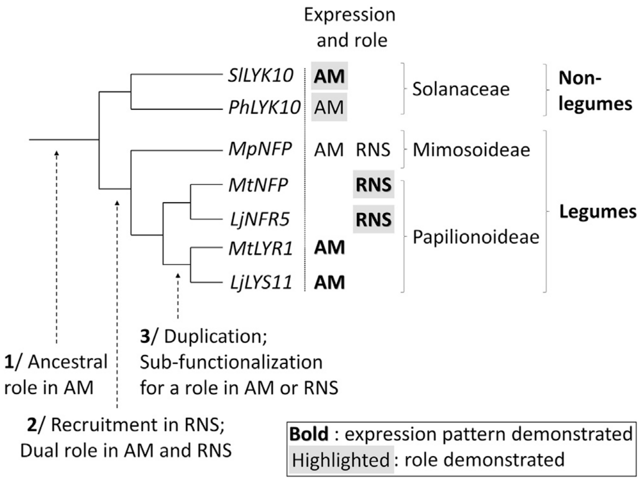

Figure 7. Proposed Scenario for Evolution of the *LYRIA* Genes

(1/) Ancestral *LYRIA* genes were involved in AM.

(2/) When the RNS appeared, *LYRIA* genes had a dual function in AM and RNS in legumes.

(3/) After gene duplication, *LYRIA* genes were sub-functionalized for a role in RNS or in AM.

Shown are putative (observed in the *M. truncatula* heterologous system) or known (bold, demonstrated in the endogenous system) expression in mycorrhizal roots (AM) and/or nodules (RNS). Putative or known (highlighted) role in AM and/or RNS are shown. *Ph*, *Petunia hybrida; Sl*, *Solananum lycopersicum* (tomato); *Mp*, *Mimosa pudica*; *Mt*, *Medicago truncatula*; and *Lj*, *Lotus japonicus*.

Indeed, Ca^{2+} spiking can be measured in an *Mtnfp* mutant after treatment with CO4 [16], suggesting that short-chain CO receptors are also involved in AM establishment. Other signals such as karrikin-like molecules and effector proteins produced by AMFs are known to play important roles in plant-AMF communication [46], but the connection of their perception and/or mode of action to LCO-mediated signaling remains elusive.

It has been postulated that RNS has evolved through recruitment of genes implicated in AM, but it is unclear how the LCO perception machinery may have been affected by the evolution of RNS. Our data are compatible with a scenario in which an ancestral *LYRIA* gene involved in LCO perception in AM was directly recruited for LCO perception for RNS in legumes (Figure 7). Because both symbiotic interfaces are intracellular, it can be proposed that *LYRIA* genes participate in these conserved accommodation mechanisms [47]. The promoters of the single *LYRIA* gene from the Solanaceae or from the legume *M. pudica* have the ability to drive dual expression both in mycorrhizal roots and in nodules of *M. truncatula*. In contrast, the *LYRIA* gene pairs in the legumes *Medicago* and *Lotus*, *MtNFP/LjNFR5*, and *MtLYR1/LjLYS11* have retained transcriptional regulation only during nodulation or AM, respectively [10, 36, 45]. This is indicative of promoter sub-functionalization following the whole genome duplication that predated the radiation of the Papilionoideae, the legume clade to which *Medicago* and *Lotus* belong (Figure 7). Interestingly, RNS is evolutionarily more stable in Papilionoideae than in any other clade of RNS-forming plants, including the Mimosoideae to which Mimosa belongs [48]. In other words, the probability for a given species in the Papilionoideae to lose RNS is much lower than in other clades. Although the reason for this greater stability remains unknown, one possibility is that duplication and sub-functionalization of genes with a dual function in AM and RNS such as the *LYRIA* genes, for separated functions in AM and RNS, may have allowed stabilized symbiotic associations.

The AAAGCTANNGACA sequence conserved in *LYRIA* promoters could represent an ancestral *cis*-regulatory element involved in transcriptional regulation during AM that has been recruited for transcriptional regulation during RNS. This putative *cis*-regulatory element is, however, conserved in the promoters of both paralogous *LYRIA* genes from the Papilionoideae, suggesting that sub-functionalization of the *LYRIA* promoter pairs has not occurred through divergence in this sequence. Further studies are required to validate the function of this putative *cis*-regulatory element and to identify the mechanism of *LYRIA* promoter sub-functionalization in Papilionoideae.

Strikingly, the Solanaceae *LYRIA* proteins PhLYK10 and SlLYK10 can restore the full nodulation program in the legume *LYRIA* mutants *Mtnfp* and *Ljnfr5*, although with lower efficiency than the respective endogenous *LYRIA* genes *MtNFP* and *LjNFR5.* This suggests that the legume and non-legume *LYRIA* proteins can fulfill the function of endogenous *LYRIA* proteins for both nodule formation and rhizobial colonization. Lower complementation efficiency of SlLYK10, PhLYK10, and PsSYM10 compared with MtNFP correlated with lower levels of protein detected in complemented *Mtnfp* roots. However, lower complementation efficiency of heterologous *LYRIA* proteins in *Mtnfp* and *Ljnfr5* may also be due to inefficient interactions with the respective co-receptors MtLYK3 and LjNFR1, two LysM-RLKs belonging the *LYKI* group. It has been suggested that evolution of the *LYRIA* gene for a new role in RNS may have involved a tandem gene duplication (preceding the advent of RNS) followed by neofunctionalization of one copy for RNS and loss of other copy in the species that acquired the RNS [49]. However, our results suggest that both the promoter and the CDS of the ancestral non-duplicated *LYRIA* gene were already fully competent for both symbioses.

Intriguingly, our results raise the question of how signal specificity in AM and RNS may be encoded. The fact that PhLYK10 can complement both *Mtnfp* and *Ljnfr5* for nodule formation while *M. truncatula* and *L. japonicus* can specifically recognize the respective major LCOs produced by *Sinorhizobium meliloti* (LCO-IV(C16:2,S) [50] and *Mesorhizobium loti* (LCO-V(C16:1,Cb,Fuc,Ac) [51] argues for limited LCO selectivity of MtNFP, LjNFR5, and their Solanaceous orthologs PhLYK10 and SlLYK10. This questions the hypothesis that MtNFP and LjNFR5 recognize specific LCO structures and suggests that co-receptors such as MtLYK3/LjNFR1, or yet unidentified proteins, may interact with MtNFP and LjNFR5 to confer LCO binding specificity to LCO receptor complexes. Consistent with such a scenario, the number of LysM-RLKs in the *LYKI* group has dramatically increased in legumes compared with non-legumes and contains a legume-specific subgroup to which *MtLYK3* and *LjNFR1* belong [52].

STAR★METHODS

Detailed methods are provided in the online version of this paper and include the following:

- KEY RESOURCES TABLE
- LEAD CONTACT AND MATERIALS AVAILABILITY

- EXPERIMENTAL MODEL AND SUBJECT DETAILS
  - Cloning
  - S. lycopersicum and P. hybrida mutant identification and genotyping
  - Agrobacterium rhizogenes mediated transformation
  - Inoculation with AMF
  - Inoculation with rhizobia and spontaneous nodulation
  - Transient Expression in N. benthamiana
- METHOD DETAILS
  - Microscopy
  - Western blotting and membrane fraction preparation
  - LCO binding assays
  - PNGaseF treatment and immunoblotting
  - qRT-PCR
  - Promoter investigation
- QUANTIFICATION AND STATISTICAL ANALYSIS
- DATA AND CODE AVAILABILITY

SUPPLEMENTAL INFORMATION

Supplemental Information can be found online at https://doi.org/10.1016/j.cub.2019.11.038.

ACKNOWLEDGMENTS

We thank Marie Cumener, Fabienne Maillet, Mireille Chabaud, and Véréna Poinsot for technical help, Sébastien Fort for CO4 an CO8 production, and Julie Cullimore and Malick Mbengue for critical reading of the manuscript. This work was supported by the ANR "WHEATSYM" (ANR-16-CE20-0025-01), the "Laboratoire d'Excellence (LABEX)" TULIP (ANR-10-LABX-41), the Swiss National Science Foundation (31003A_169732), and the research project Engineering Nitrogen Symbiosis for Africa (ENSA), which is funded through a grant to the University of Cambridge by the Bill & Melinda Gates Foundation (OPP1172165). A.G.'s fellowship was funded by Région Occitanie and INRA Department of Plant Health and Environment (SPE). T.W.'s fellowship was funded by the Chinese Scholarship Council (CSC). Work in M.P.'s lab was funded by the ERC Advanced Grant ERC-2013-ADG: "Molecular inventions underlying the evolution of the nitrogen-fixing root nodule symbiosis" (EVOLVINGNODULES).

AUTHOR CONTRIBUTIONS

Conceptualization, B.L., D.R., J.-J.B., M.P., and P.-M.D.; Investigation, A.G., C.R., J.K., L.B., M.-C.A., M.G., M.R., T.V., T.W., V.G., and Y.D.; Resources, A.B., M.S., M.V., and P.M.; Writing, A.G., B.L., D.R., J.-J.B., M.P., and P.-M.D.

DECLARATION OF INTERESTS

The authors declare no competing interests.

Received: February 22, 2019
Revised: August 9, 2019
Accepted: November 12, 2019
Published: December 5, 2019

REFERENCES

1. Delaux, P.M., Radhakrishnan, G.V., Jayaraman, D., Cheema, J., Malbreil, M., Volkening, J.D., Sekimoto, H., Nishiyama, T., Melkonian, M., Pokorny, L., et al. (2015). Algal ancestor of land plants was preadapted for symbiosis. Proc. Natl. Acad. Sci. USA *112*, 13390–13395.
2. Rich, M.K., Nouri, E., Courty, P.E., and Reinhardt, D. (2017). Diet of Arbuscular Mycorrhizal Fungi: Bread and Butter? Trends Plant Sci. *22*, 652–660.
3. Parniske, M. (2008). Arbuscular mycorrhiza: the mother of plant root endosymbioses. Nat. Rev. Microbiol. *6*, 763–775.
4. Murray, J.D. (2011). Invasion by invitation: rhizobial infection in legumes. Mol. Plant Microbe Interact. *24*, 631–639.
5. Fliegmann, J., and Bono, J.J. (2015). Lipo-chitooligosaccharidic nodulation factors and their perception by plant receptors. Glycoconj. J. *32*, 455–464.
6. Fliegmann, J., Canova, S., Lachaud, C., Uhlenbroich, S., Gasciolli, V., Pichereaux, C., Rossignol, M., Rosenberg, C., Cumener, M., Pitorre, D., et al. (2013). Lipo-chitooligosaccharidic symbiotic signals are recognized by LysM receptor-like kinase LYR3 in the legume Medicago truncatula. ACS Chem. Biol. *8*, 1900–1906.
7. Malkov, N., Fliegmann, J., Rosenberg, C., Gasciolli, V., Timmers, A.C., Nurisso, A., Cullimore, J., and Bono, J.J. (2016). Molecular basis of lipo-chitooligosaccharide recognition by the lysin motif receptor-like kinase LYR3 in legumes. Biochem. J. *473*, 1369–1378.
8. Broghammer, A., Krusell, L., Blaise, M., Sauer, J., Sullivan, J.T., Maolanon, N., Vinther, M., Lorentzen, A., Madsen, E.B., Jensen, K.J., et al. (2012). Legume receptors perceive the rhizobial lipochitin oligosaccharide signal molecules by direct binding. Proc. Natl. Acad. Sci. USA *109*, 13859–13864.
9. Buendia, L., Girardin, A., Wang, T., Cottret, L., and Lefebvre, B. (2018). LysM Receptor-Like Kinase and LysM Receptor-Like Protein Families: An Update on Phylogeny and Functional Characterization. Front. Plant Sci. *9*, 1531.
10. Arrighi, J.F., Barre, A., Ben Amor, B., Bersoult, A., Soriano, L.C., Mirabella, R., de Carvalho-Niebel, F., Journet, E.P., Ghérardi, M., Huguet, T., et al. (2006). The Medicago truncatula lysin [corrected] motif-receptor-like kinase gene family includes NFP and new nodule-expressed genes. Plant Physiol. *142*, 265–279.
11. Radutoiu, S., Madsen, L.H., Madsen, E.B., Felle, H.H., Umehara, Y., Grønlund, M., Sato, S., Nakamura, Y., Tabata, S., Sandal, N., and Stougaard, J. (2003). Plant recognition of symbiotic bacteria requires two LysM receptor-like kinases. Nature *425*, 585–592.
12. Indrasumunar, A., Kereszt, A., Searle, I., Miyagi, M., Li, D., Nguyen, C.D., Men, A., Carroll, B.J., and Gresshoff, P.M. (2010). Inactivation of duplicated nod factor receptor 5 (NFR5) genes in recessive loss-of-function non-nodulation mutants of allotetraploid soybean (Glycine max L. Merr.). Plant Cell Physiol. *51*, 201–214.
13. Maillet, F., Poinsot, V., André, O., Puech-Pagès, V., Haouy, A., Gueunier, M., Cromer, L., Giraudet, D., Formey, D., Niebel, A., et al. (2011). Fungal lipochitooligosaccharide symbiotic signals in arbuscular mycorrhiza. Nature *469*, 58–63.
14. Sun, J., Miller, J.B., Granqvist, E., Wiley-Kalil, A., Gobbato, E., Maillet, F., Cottaz, S., Samain, E., Venkateshwaran, M., Fort, S., et al. (2015). Activation of symbiosis signaling by arbuscular mycorrhizal fungi in legumes and rice. Plant Cell *27*, 823–838.
15. Camps, C., Jardinaud, M.F., Rengel, D., Carrère, S., Hervé, C., Debellé, F., Gamas, P., Bensmihen, S., and Gough, C. (2015). Combined genetic and transcriptomic analysis reveals three major signalling pathways activated by Myc-LCOs in Medicago truncatula. New Phytol. *208*, 224–240.
16. Genre, A., Chabaud, M., Balzergue, C., Puech-Pagès, V., Novero, M., Rey, T., Fournier, J., Rochange, S., Bécard, G., Bonfante, P., and Barker, D.G. (2013). Short-chain chitin oligomers from arbuscular mycorrhizal fungi trigger nuclear Ca2+ spiking in Medicago truncatula roots and their production is enhanced by strigolactone. New Phytol. *198*, 190–202.
17. Op den Camp, R., Streng, A., De Mita, S., Cao, Q., Polone, E., Liu, W., Ammiraju, J.S., Kudrna, D., Wing, R., Untergasser, A., et al. (2011). LysM-type mycorrhizal receptor recruited for rhizobium symbiosis in nonlegume Parasponia. Science *331*, 909–912.
18. Miyata, K., Hayafune, M., Kobae, Y., Kaku, H., Nishizawa, Y., Masuda, Y., Shibuya, N., and Nakagawa, T. (2016). Evaluation of the Role of the LysM Receptor-Like Kinase, OsNFR5/OsRLK2 for AM Symbiosis in Rice. Plant Cell Physiol. *57*, 2283–2290.

19. Zhang, X., Dong, W., Sun, J., Feng, F., Deng, Y., He, Z., Oldroyd, G.E., and Wang, E. (2015). The receptor kinase CERK1 has dual functions in symbiosis and immunity signalling. Plant J. *81*, 258–267.

20. Miyata, K., Kozaki, T., Kouzai, Y., Ozawa, K., Ishii, K., Asamizu, E., Okabe, Y., Umehara, Y., Miyamoto, A., Kobae, Y., et al. (2014). The bifunctional plant receptor, OsCERK1, regulates both chitin-triggered immunity and arbuscular mycorrhizal symbiosis in rice. Plant Cell Physiol. *55*, 1864–1872.

21. Buendia, L., Wang, T., Girardin, A., and Lefebvre, B. (2016). The LysM receptor-like kinase SlLYK10 regulates the arbuscular mycorrhizal symbiosis in tomato. New Phytol. *210*, 184–195.

22. Liao, D., Sun, X., Wang, N., Song, F., and Liang, Y. (2018). Tomato LysM Receptor-Like Kinase SlLYK12 Is Involved in Arbuscular Mycorrhizal Symbiosis. Front. Plant Sci. *9*, 1004.

23. Gibelin-Viala, C., Amblard, E., Puech-Pages, V., Bonhomme, M., Garcia, M., Bascaules-Bedin, A., Fliegmann, J., Wen, J., Mysore, K.S., le Signor, C., et al. (2019). The Medicago truncatula LysM receptor-like kinase LYK9 plays a dual role in immunity and the arbuscular mycorrhizal symbiosis. New Phytol. *223*, 1516–1529.

24. Shimizu, T., Nakano, T., Takamizawa, D., Desaki, Y., Ishii-Minami, N., Nishizawa, Y., Minami, E., Okada, K., Yamane, H., Kaku, H., and Shibuya, N. (2010). Two LysM receptor molecules, CEBiP and OsCERK1, cooperatively regulate chitin elicitor signaling in rice. Plant J. *64*, 204–214.

25. Ao, Y., Li, Z., Feng, D., Xiong, F., Liu, J., Li, J.F., Wang, M., Wang, J., Liu, B., and Wang, H.B. (2014). OsCERK1 and OsRLCK176 play important roles in peptidoglycan and chitin signaling in rice innate immunity. Plant J. *80*, 1072–1084.

26. Carotenuto, G., Chabaud, M., Miyata, K., Capozzi, M., Takeda, N., Kaku, H., Shibuya, N., Nakagawa, T., Barker, D.G., and Genre, A. (2017). The rice LysM receptor-like kinase OsCERK1 is required for the perception of short-chain chitin oligomers in arbuscular mycorrhizal signaling. New Phytol. *214*, 1440–1446.

27. Bravo, A., York, T., Pumplin, N., Mueller, L.A., and Harrison, M.J. (2016). Genes conserved for arbuscular mycorrhizal symbiosis identified through phylogenomics. Nat. Plants *2*, 15208.

28. Delaux, P.M., Varala, K., Edger, P.P., Coruzzi, G.M., Pires, J.C., and Ané, J.M. (2014). Comparative phylogenomics uncovers the impact of symbiotic associations on host genome evolution. PLoS Genet. *10*, e1004487.

29. Vandenbussche, M., Janssen, A., Zethof, J., van Orsouw, N., Peters, J., van Eijk, M.J., Rijpkema, A.S., Schneiders, H., Santhanam, P., de Been, M., et al. (2008). Generation of a 3D indexed Petunia insertion database for reverse genetics. Plant J. *54*, 1105–1114.

30. Lefebvre, B., Klaus-Heisen, D., Pietraszewska-Bogiel, A., Hervé, C., Camut, S., Auriac, M.-C., Gasciolli, V., Nurisso, A., Gadella, T.W.J., and Cullimore, J. (2012). Role of N-glycosylation sites and CXC motifs in trafficking of medicago truncatula Nod factor perception protein to plasma membrane. J. Biol. Chem. *287*, 10812–10823.

31. Kawaharada, Y., Kelly, S., Nielsen, M.W., Hjuler, C.T., Gysel, K., Muszyński, A., Carlson, R.W., Thygesen, M.B., Sandal, N., Asmussen, M.H., et al. (2015). Receptor-mediated exopolysaccharide perception controls bacterial infection. Nature *523*, 308–312.

32. Carroll, S.B. (2008). Evo-devo and an expanding evolutionary synthesis: a genetic theory of morphological evolution. Cell *134*, 25–36.

33. Jiang, P., and Rausher, M. (2018). Two genetic changes in cis-regulatory elements caused evolution of petal spot position in Clarkia. Nat. Plants *4*, 14–22.

34. Gutjahr, C., and Paszkowski, U. (2013). Multiple control levels of root system remodeling in arbuscular mycorrhizal symbiosis. Front. Plant Sci. *4*, 204.

35. Kobae, Y., and Hata, S. (2010). Dynamics of periarbuscular membranes visualized with a fluorescent phosphate transporter in arbuscular mycorrhizal roots of rice. Plant Cell Physiol. *51*, 341–353.

36. Gomez, S.K., Javot, H., Deewatthanawong, P., Torres-Jerez, I., Tang, Y., Blancaflor, E.B., Udvardi, M.K., and Harrison, M.J. (2009). Medicago truncatula and Glomus intraradices gene expression in cortical cells harboring arbuscules in the arbuscular mycorrhizal symbiosis. BMC Plant Biol. *9*, 10.

37. Griesmann, M., Chang, Y., Liu, X., Song, Y., Haberer, G., Crook, M.B., Billault-Penneteau, B., Lauressergues, D., Keller, J., Imanishi, L., et al. (2018). Phylogenomics reveals multiple losses of nitrogen-fixing root nodule symbiosis. Science *361*, eaat1743.

38. Monte, I., Ishida, S., Zamarreño, A.M., Hamberg, M., Franco-Zorrilla, J.M., García-Casado, G., Gouhier-Darimont, C., Reymond, P., Takahashi, K., García-Mina, J.M., et al. (2018). Ligand-receptor co-evolution shaped the jasmonate pathway in land plants. Nat. Chem. Biol. *14*, 480–488.

39. Bensmihen, S., de Billy, F., and Gough, C. (2011). Contribution of NFP LysM domains to the recognition of Nod factors during the Medicago truncatula/Sinorhizobium meliloti symbiosis. PLoS ONE *6*, e26114.

40. Ried, M.K., Antolín-Llovera, M., and Parniske, M. (2014). Spontaneous symbiotic reprogramming of plant roots triggered by receptor-like kinases. eLife *3*.

41. Czaja, L.F., Hogekamp, C., Lamm, P., Maillet, F., Martinez, E.A., Samain, E., Dénarié, J., Küster, H., and Hohnjec, N. (2012). Transcriptional responses toward diffusible signals from symbiotic microbes reveal MtNFP- and MtDMI3-dependent reprogramming of host gene expression by arbuscular mycorrhizal fungal lipochitooligosaccharides. Plant Physiol. *159*, 1671–1685.

42. Buendia, L., Maillet, F., O'Connor, D., van de-Kerkhove, Q., Danoun, S., Gough, C., Lefebvre, B., and Bensmihen, S. (2019). Lipo-chitooligosaccharides promote lateral root formation and modify auxin homeostasis in Brachypodium distachyon. New Phytol. *221*, 2190–2202.

43. Amor, B.B., Shaw, S.L., Oldroyd, G.E.D., Maillet, F., Penmetsa, R.V., Cook, D., Long, S.R., Dénarié, J., and Gough, C. (2003). The NFP locus of Medicago truncatula controls an early step of Nod factor signal transduction upstream of a rapid calcium flux and root hair deformation. Plant J. *34*, 495–506.

44. Hohnjec, N., Czaja-Hasse, L.F., Hogekamp, C., and Küster, H. (2015). Pre-announcement of symbiotic guests: transcriptional reprogramming by mycorrhizal lipochitooligosaccharides shows a strict co-dependency on the GRAS transcription factors NSP1 and RAM1. BMC Genomics *16*, 994.

45. Rasmussen, S.R., Füchtbauer, W., Novero, M., Volpe, V., Malkov, N., Genre, A., Bonfante, P., Stougaard, J., and Radutoiu, S. (2016). Intraradical colonization by arbuscular mycorrhizal fungi triggers induction of a lipochitooligosaccharide receptor. Sci. Rep. *6*, 29733.

46. Lanfranco, L., Fiorilli, V., and Gutjahr, C. (2018). Partner communication and role of nutrients in the arbuscular mycorrhizal symbiosis. New Phytol. *220*, 1031–1046.

47. Parniske, M. (2000). Intracellular accommodation of microbes by plants: a common developmental program for symbiosis and disease? Curr. Opin. Plant Biol. *3*, 320–328.

48. Werner, G.D., Cornwell, W.K., Sprent, J.I., Kattge, J., and Kiers, E.T. (2014). A single evolutionary innovation drives the deep evolution of symbiotic N2-fixation in angiosperms. Nat. Commun. *5*, 4087.

49. van Velzen, R., Holmer, R., Bu, F., Rutten, L., van Zeijl, A., Liu, W., Santuari, L., Cao, Q., Sharma, T., Shen, D., et al. (2018). Comparative genomics of the nonlegume *Parasponia* reveals insights into evolution of nitrogen-fixing rhizobium symbioses. Proc. Natl. Acad. Sci. USA *115*, E4700–E4709.

50. Lerouge, P., Roche, P., Faucher, C., Maillet, F., Truchet, G., Promé, J.C., and Dénarié, J. (1990). Symbiotic host-specificity of Rhizobium meliloti is determined by a sulphated and acylated glucosamine oligosaccharide signal. Nature *344*, 781–784.

51. Bek, A.S., Sauer, J., Thygesen, M.B., Duus, J.Ø., Petersen, B.O., Thirup, S., James, E., Jensen, K.J., Stougaard, J., and Radutoiu, S. (2010). Improved characterization of nod factors and genetically based variation in LysM Receptor domains identify amino acids expendable for nod factor recognition in Lotus spp. Mol. Plant Microbe Interact. *23*, 58–66.

52. De Mita, S., Streng, A., Bisseling, T., and Geurts, R. (2014). Evolution of a symbiotic receptor through gene duplications in the legume-rhizobium mutualism. New Phytol. *201*, 961–972.
53. Maudoux, O., Batoko, H., Oecking, C., Gevaert, K., Vandekerckhove, J., Boutry, M., and Morsomme, P. (2000). A plant plasma membrane H^+-ATPase expressed in yeast is activated by phosphorylation at its penultimate residue and binding of 14-3-3 regulatory proteins in the absence of fusicoccin. J. Biol. Chem. *275*, 17762–17770.
54. Quandt, H.J., Puhler, A., and Broer, I. (1993). Transgenic root-nodules of V*icia-hirsuta*: a fast and efficient system for the study of gene-expression in indeterminate-type nodules. Mol. Plant Microbe Interact. *6*, 699–706.
55. Ardourel, M., Demont, N., Debellé, F., Maillet, F., de Billy, F., Promé, J.C., Dénarié, J., and Truchet, G. (1994). *Rhizobium meliloti* lipooligosaccharide nodulation factors: different structural requirements for bacterial entry into target root hair cells and induction of plant symbiotic developmental responses. Plant Cell *6*, 1357–1374.
56. Maekawa-Yoshikawa, M., Müller, J., Takeda, N., Maekawa, T., Sato, S., Tabata, S., Perry, J., Wang, T.L., Groth, M., Brachmann, A., and Parniske, M. (2009). The temperature-sensitive brush mutant of the legume Lotus japonicus reveals a link between root development and nodule infection by rhizobia. Plant Physiol. *149*, 1785–1796.
57. van der Fits, L., Deakin, E.A., Hoge, J.H.C., and Memelink, J. (2000). The ternary transformation system: constitutive virG on a compatible plasmid dramatically increases Agrobacterium-mediated plant transformation. Plant Mol. Biol. *43*, 495–502.
58. Sejalon-Delmas, N., Magnier, A., Douds, D., and Becard, G. (1998). Cytoplasmic autofluorescence of an arbuscular mycorrhizal fungus Gigaspora gigantea and nondestructive fungal observations in planta. Mycologia *90*, 921–926.
59. Poupot, R., Martinez-Romero, E., and Promé, J.C. (1993). Nodulation factors from Rhizobium tropici are sulfated or nonsulfated chitopentasaccharides containing an N-methyl-N-acylglucosaminyl terminus. Biochemistry *32*, 10430–10435.
60. Stuurman, J., and Kuhlemeier, C. (2005). Stable two-element control of dTph1 transposition in mutator strains of Petunia by an inactive ACT1 introgression from a wild species. Plant J. *41*, 945–955.
61. Madsen, E.B., Madsen, L.H., Radutoiu, S., Olbryt, M., Rakwalska, M., Szczyglowski, K., Sato, S., Kaneko, T., Tabata, S., Sandal, N., and Stougaard, J. (2003). A receptor kinase gene of the LysM type is involved in legume perception of rhizobial signals. Nature *425*, 637–640.
62. Pietraszewska-Bogiel, A., Lefebvre, B., Koini, M.A., Klaus-Heisen, D., Takken, F.L.W., Geurts, R., Cullimore, J.V., and Gadella, T.W.J. (2013). Interaction of Medicago truncatula lysin motif receptor-like kinases, NFP and LYK3, produced in Nicotiana benthamiana induces defence-like responses. PLoS ONE *8*, e65055.
63. Kirienko, A.N., Porozov, Y.B., Malkov, N.V., Akhtemova, G.A., Le Signor, C., Thompson, R., Saffray, C., Dalmais, M., Bendahmane, A., Tikhonovich, I.A., and Dolgikh, E.A. (2018). Role of a receptor-like kinase K1 in pea Rhizobium symbiosis development. Planta *248*, 1101–1120.
64. Lefebvre, B., Batoko, H., Duby, G., and Boutry, M. (2004). Targeting of a Nicotiana plumbaginifolia H+ -ATPase to the plasma membrane is not by default and requires cytosolic structural determinants. Plant Cell *16*, 1772–1789.
65. Camacho, C., Coulouris, G., Avagyan, V., Ma, N., Papadopoulos, J., Bealer, K., and Madden, T.L. (2009). BLAST+: architecture and applications. BMC Bioinformatics *10*, 421.
66. Katoh, K., and Standley, D.M. (2013). MAFFT multiple sequence alignment software version 7: improvements in performance and usability. Mol. Biol. Evol. *30*, 772–780.
67. Capella-Gutiérrez, S., Silla-Martínez, J.M., and Gabaldón, T. (2009). trimAl: a tool for automated alignment trimming in large-scale phylogenetic analyses. Bioinformatics *25*, 1972–1973.
68. Kalyaanamoorthy, S., Minh, B.Q., Wong, T.K.F., von Haeseler, A., and Jermiin, L.S. (2017). ModelFinder: fast model selection for accurate phylogenetic estimates. Nat. Methods *14*, 587–589.
69. Nguyen, L.T., Schmidt, H.A., von Haeseler, A., and Minh, B.Q. (2015). IQ-TREE: a fast and effective stochastic algorithm for estimating maximum-likelihood phylogenies. Mol. Biol. Evol. *32*, 268–274.
70. Guindon, S., Dufayard, J.F., Lefort, V., Anisimova, M., Hordijk, W., and Gascuel, O. (2010). New algorithms and methods to estimate maximum-likelihood phylogenies: assessing the performance of PhyML 3.0. Syst. Biol. *59*, 307–321.
71. Letunic, I., and Bork, P. (2016). Interactive tree of life (iTOL) v3: an online tool for the display and annotation of phylogenetic and other trees. Nucleic Acids Res. *44* (W1), W242–245.
72. Bailey, T.L., and Elkan, C. (1994). Fitting a mixture model by expectation maximization to discover motifs in biopolymers. Proc. Int. Conf. Intell. Syst. Mol. Biol. *2*, 28–36.
73. Sevin-Pujol, A., Sicard, M., Rosenberg, C., Auriac, M.C., Lepage, A., Niebel, A., Gough, C., and Bensmihen, S. (2017). Development of a GAL4-VP16/UAS trans-activation system for tissue specific expression in Medicago truncatula. PLoS ONE *12*, e0188923.
74. Lefebvre, B., Timmers, T., Mbengue, M., Moreau, S., Hervé, C., Tóth, K., Bittencourt-Silvestre, J., Klaus, D., Deslandes, L., Godiard, L., et al. (2010). A remorin protein interacts with symbiotic receptors and regulates bacterial infection. Proc. Natl. Acad. Sci. USA *107*, 2343–2348.
75. Binder, A., Lambert, J., Morbitzer, R., Popp, C., Ott, T., Lahaye, T., and Parniske, M. (2014). A modular plasmid assembly kit for multigene expression, gene silencing and silencing rescue in plants. PLoS ONE *9*, e88218.
76. Chabaud, M., Boisson-Dernier, A., Zhang, J., Taylor, C.G., Yu, O., and Barker, D.G. (2006). Agrobacterium rhizogenes-mediated root transformation. In The Medicago truncatula handbook, U. Mathesius,, E.P. Journet, and L.W. Sumner, eds. (Noble Research Institute), pp. 1–8.
77. Charpentier, M., Bredemeier, R., Wanner, G., Takeda, N., Schleiff, E., and Parniske, M. (2008). Lotus japonicus CASTOR and POLLUX are ion channels essential for perinuclear calcium spiking in legume root endosymbiosis. Plant Cell *20*, 3467–3479.
78. Becard, G., and Fortin, J.A. (1988). Early events of vesicular-arbuscular mycorrhiza formation on Ri T-DNA transformed roots. New Phytol. *108*, 211–218.
79. Mbengue, M., Camut, S., de Carvalho-Niebel, F., Deslandes, L., Froidure, S., Klaus-Heisen, D., Moreau, S., Rivas, S., Timmers, T., Hervé, C., et al. (2010). The Medicago truncatula E3 ubiquitin ligase PUB1 interacts with the LYK3 symbiotic receptor and negatively regulates infection and nodulation. Plant Cell *22*, 3474–3488.
80. Gressent, F., Cullimore, J.V., Ranjeva, R., and Bono, J.J. (2004). Radiolabeling of lipo-chitooligosaccharides using the NodH sulfotransferase: a two-step enzymatic procedure. BMC Biochem. *5*, 4.
81. Rich, M.K., Schorderet, M., Bapaume, L., Falquet, L., Morel, P., Vandenbussche, M., and Reinhardt, D. (2015). The Petunia GRAS Transcription Factor ATA/RAM1 Regulates Symbiotic Gene Expression and Fungal Morphogenesis in Arbuscular Mycorrhiza. Plant Physiol. *168*, 788–797.

STAR★METHODS

KEY RESOURCES TABLE

REAGENT or RESOURCE	SOURCE	IDENTIFIER
Antibodies		
Rabbit polyclonal GFP antibodies	AMSBIO	TP401; RRID: AB_10890443
monoclonal HSC70 (BIP) antibody	Enzo Life Sciences	ADI-SPA-818; RRID: AB_10617235
Rabbit polyclonal H^+-ATPase antibodies	[53]	N/A
Fungal and Bacterial Strains		
Agrobacterium rhizogenes ARquA1	[54]	N/A
Sinorhizobium meliloti 2011 pXLGD4 (lacZ reporter)	[55]	N/A
Mesorhizobium loti MAFF303099 *Ds*RED	[56]	N/A
Agrobacterium tumefaciens LBA4404 VirGN54D	[57]	N/A
Rhizophagus irregularis DAOM 197198	Agronutrition	AP2007-A
Gigaspora gigantea	[58]	N/A
Chemicals, Peptides, and Recombinant Proteins		
FM4-64	Invitrogen	T3320
DAPI	SIGMA	D9542
PNGaseF	Roche Diagnostics	11365169001
LCO-V(C18:1Δ11,NMe) purified from *Rhizobium tropici*	[59]	N/A
LCO-V(C18:1Δ11,NMe,S) purified from *Rhizobium tropici*	[59]	N/A
Myc-LCOs, LCO-IV(C18:1Δ9)	[13]	N/A
LCO-IV(C18:1Δ9,S)	[13]	N/A
LCO-IV(C16:0)	[13]	N/A
LCO-IV(C16:O,S)	[13]	N/A
CO4	CERMAV Grenoble, France	N/A
CO8	CERMAV Grenoble, France	N/A
Critical Commercial Assays		
Gateway BP mix	Invitrogen	11789-100
Gateway LR mix	Invitrogen	11791-100
Bsa I enzyme for Golden Gate reactions	New England BIOLABS	R0535S
X-Gluc	Biosynth	B7300
Magenta-Gluc	Biosynth	B7350
X-Gal substrate	Thermofisher	10113253
WGA CF488A conjugate	Biotum	BTM29022
Macherey-Nagel NUCLEOSPIN RNA kit	Macherey-Nagel	740955.250
Agilent RNA Nano Chip and Reagents	Agilent Technologies	5067-1511
Superscript reverse transcriptase	Invitrogen	18064071
LightCycler480 Sybr Green I Master	Roche	04707516001
Attapulgite (American granules plain)	Oil-dri UK	GB100
Experimental Models: Organisms/Strains		
Solanum lycopersicum cv Marmande	NA	N/A
Petunia hybrida cv W138	NA	N/A
Petunia hybrida cv W5	[60]	N/A
Mimosa pudica	[37]	N/A
Solanum lycopersicum cv M82 *Sllyk10-1*	This work	N/A
Petunia hybrida cv W138 *Phlyk10-1*	This work	N/A
Medicago truncatula A17	NA	N/A
Medicago truncatula A17 *Mtnfp-2*	[10]	N/A

(*Continued on next page*)

Continued

REAGENT or RESOURCE	SOURCE	IDENTIFIER
Lotus japonicus Gifu	NA	N/A
Lotus japonicus Ljnfr5-2	[61]	N/A
Oligonucleotides		
ProSlLYK10 for GGTCTCTAAATGGGTTATAGAGCTGTAATGC	This work	N/A
ProSlLYK10 rev GGTCTCATTTGCGATGCAAAGCTTAGATAAC	This work	N/A
ProPhLYK10 for ATCGGTCTCCAAATGAGCTGCAGGGCTTTTCTACG	This work	N/A
ProPhLYK10 rev ATCGGTCTCCTTTGTGCTGCAAAGCTCAGATGGC	This work	N/A
ProMpNFP for ATCGGTCTCCAAATAGAAAGTTTTCTGTTGTCCGG	This work	N/A
ProMpNFP rev: ATCGGTCTCCTTTGCTAATGAGAGTTTAGCAGAGG	This work	N/A
PhLYK10ECR for: GGTCTCCCAAAATGGTAGCTCCTCTTGCCTCCT	This work	N/A
PhLYK10ECR rev: GGTCTCGTAAGAATACTTAAAACGACAATGAGA	This work	N/A
SlLYK10ECR for GGTCTCGCAAAATGGTAGTTCCTCTTGTGTCCTTG	This work	N/A
SlLYK10ECR rev GGTCTCGTAAGTCCATGCTTGGATTTTCTACTGCTTGC	This work	N/A
SlLYK10 Genotyping for: GTGGTGCAAGATATGAATCC	This work	N/A
SlLYK10 Genotyping rev: GAGCTAAGTTAGACCTCCTC	This work	N/A
PhLYK10 Genotyping for: GCAGACAGAGACTTTTTGTGCTCT	This work	N/A
PhLYK10 Genotyping rev: ACAGCTTCCGTACCAACTGTC	This work	N/A
Recombinant DNA		
Pcambia-Pro35S:PhLYK10-YFP	This work	N/A
PcambiaGG-Pro35S:PhLYK10c-YFP	This work	N/A
PcambiaGG-Pro35S:SlLYK10-YFP	This work	N/A
PcambiaGG-Pro35S:SlLYK10c-YFP	This work	N/A
PbinGW-Pro35S:SlLYK10-YFP	This work	N/A
PcambiaGG-ProSlLYK10:GUS	This work	N/A
PcambiaGG-ProminSlLYK10:GUS	This work	N/A
PcambiaGG-ProPhLYK10:GUS	This work	N/A
PcambiaGG-ProMpNFP:GUS	This work	N/A
Pbin-ProMtNFP:GUS	[10]	N/A
PcambiaGG-ProminMtNFP:GUS	This work	N/A
Pbin-ProMtNFP:MtNFP-YFP	This work	N/A
PcambiaGG-ProSlLYK10:MtNFP-YFP	This work	N/A
Pcambia-Pro35S:AtCERK1-YFP	[62]	N/A
Pcambia-ProLjUBI:LjNFR5-mOrange	This work	N/A
Pcambia-ProLjUBI:PhLYK10-mOrange	This work	N/A
Pbin-PsSYM10-YFP	[63]	N/A
Pbin-pro35S:PMA4-GFP	[64]	N/A
Pbin-pro35S:HDEL-GFP	[64]	N/A
Software and Algorithms		
LASX	Leica	N/A
Zen	Leica	N/A
ImageJ	http://imagej.nih.gov/ij	N/A
R	http://r-project.org	N/A
tBLASTn v2.9.0+	[65]	N/A
MAFFT v7.407	[66]	N/A
TrimAl v1.4	[67]	N/A
ModelFinder	[68]	N/A
IQ-TREE v1.6.1	[69]	N/A
SH-alrt	[70]	N/A
iTOL platform v4.4.2	[71]	N/A

(*Continued on next page*)

Continued		
REAGENT or RESOURCE	SOURCE	IDENTIFIER
MEME v5.0.1	[72]	N/A
Other		
Medicago truncatula Gene Expression Atlas	http://mtgea.noble.org/v3	N/A
Axiozoom V16 microscope	Zeiss	N/A
Axioplan 2 microscope	Zeiss	N/A
SP2 confocal microscope	Leica	N/A
SP8 confocal microscope	Leica	N/A
S6E microscope	Leica	N/A
vribratome VT 1000S	Leica	N/A

LEAD CONTACT AND MATERIALS AVAILABILITY

Further information and requests for resources and reagents should be directed to and will be fulfilled by the Lead Contact, Benoit Lefebvre (benoit.lefebvre@inra.fr). All unique/stable reagents generated in this study are available from the Lead Contact without restriction.

EXPERIMENTAL MODEL AND SUBJECT DETAILS

Cloning

1.8, 1.5 and 1.9 kbp corresponding to the non-coding region between *SlLYK10, PhLYK10, and MpNFP* and the preceding genes, including the *5′ UTR* were amplified by PCR (with the primers listed in the key resources table) from genomic DNA isolated from *S. lycopersicum*, *P. hybrida* and *M. pudica,* respectively, and cloned in transcriptional fusion with a *GUS* reporter containing a plant intron, in a pCambia 2200 modified for Golden gate cloning and containing a *ProUbi:DsRed* reporter as in [73]. Note that the *PhLYK10* sequence in *P. hybrida* originates from the *P. axillaris* parent. 240 and 185 bp sequences preceding the *SlLYK10* or *MtNFP* start codons were synthesized and cloned as described previously. *ProSlLYK10:MtNFP-YFP* was made by Golden gate cloning in a pCambia 2200 modified for Golden gate as in [6]. *ProMtNFP:MtNFP-YFP* was made as in [30] excepted that MtNFP was in translation fusion with *YFP* instead that of *FLAG*.

SlLYK10 and *PhLYK10* coding sequences were amplified by PCR from genomic DNA isolated from *S. lycopersicum* and *P. hybrida* respectively and cloned in translational fusion with *YFP* under the control of *Pro35S* in a pbin vector modified for gateway cloning as in [74] for *SlLYK10* or in a pCambia 2200 modified for Golden gate cloning as in [6] for *PhLYK10*. For expression in *M. truncatula*, *SlLYK10* sequence was optimized with a *M. truncatula* codon usage and cloned in translational fusion with *YFP* under the control of *Pro35S* in a pCambia 2200 modified for Golden gate cloning as in [6]. For expression in *L. japonicus*, *PhLYK10* coding sequence was amplified by PCR from genomic DNA isolated from *P. hybrida* and cloned in translational fusion with *mOrange* under the control of *LjUbiquitin* promoter into a pCambia-based Golden Gate expression vector [75].

For *SlLYK10c* and *PhLYK10c* constructs, the sequences coding the extracellular region of *SlLYK10* or *PhLYK10* were amplified by PCR (with the primers listed in the key resources table) and cloned in translational fusion with the sequences coding TM/ICR of MtNFP and YFP under the control of *Pro35S* in a pCambia 2200 modified for Golden gate cloning as in [6].

S. lycopersicum and P. hybrida mutant identification and genotyping

The *Sllyk10-1* mutant allele (line 1051, $G^{460}A$) was identified by sequencing (NGS) an amplicon (key resources table) obtained on tomato (cv M82) EMS-mutagenized lines. Homozygous mutant or WT *SlLYK10* alleles were identified by sequencing (Sanger) a similar amplicon on the progeny. The *Phlyk10-1* mutant allele (line LY0882, *dTph1* insertion 116 bp from the start codon) was identified by BLAST-searching in a *Petunia dTPh1* transposon flanking sequence database [29] with the full *PhLYK10* coding sequence. This line was crossed with the stabilizer line W5 [60], to segregate out the activator locus required for *dTph1* transposition. Genotyping on different progenies was done by PCR with the primers listed in the key resources table.

Agrobacterium rhizogenes mediated transformation

Tomato (cv Marmande) seeds were surface sterilized and germinated *in vitro* for 7 to 10 days until cotyledons were fully expanded. Plantlets were cut at the hypocotyl level, immerged in a *A. rhizogenes* ARqua1 suspension at OD_{600nm} = 0.3 and grown for 3 days at 25°C on MS, then on MS supplemented with 50 mg/l kanamycin and 200 mg/l cefotaxim until emergence of transgenic roots. Transgenic roots were selected by fluorescence microscopy. Plantlets were transferred in pots containing vermiculite as described in [21]. ROC lines derived from transformed roots were grown in dark, on MS medium supplemented with 50 mg/l kanamycin.

Chimeric *M. truncatula* A17 and *Mtnfp-2* plants were produced as described in [76] for analysis of promoter expression pattern and for complementation experiment, respectively. Chimeric *L. japonicus* Gifu and *Ljnfr5-2* plants were produced as described in [77].

Inoculation with AMF

For AM phenotyping, petunia seeds were germinated on a sterilized potting soil until cotyledons were fully expanded. Tomato seeds were surface sterilized and germinated in sterile water. Petunia and tomato plantlets were then transferred in 50 mL containers filled with attapulgite, watered with 20 mL of 0.5x modified Long ashton (7.5 μM NaH_2PO_4), and inoculated with 500 spores of *R. irregularis* DAOM 197198. Roots were harvested, washed and stained between 3 and 4 weeks post inoculation.

For analysis of GUS activity in tomato roots, sterilized *Gigantea gigaspora* spores, harvested from a leek nurse culture, were pre-germinated 5 days on M medium [78] in a 3% CO_2 incubator at 32°C. Two spores and one fragment of a transgenic tomato ROC line were then co-cultured on a Petri dish containing M medium supplemented with 50 mg/l kanamycin. Petri dishes were placed vertically with ROC lines above the fungal spores for 4 weeks. For analysis of GUS activity in *M. truncatula* transgenic roots, chimeric plantlets were transferred in 50 mL containers filled with a mix 1:1 of attapulgite and sand, watered with 20 mL of 0.5x modified Long ashton medium and inoculated with 200 spores of *R. irregularis* DAOM 197198. Roots were harvested, washed and stained 2 weeks post inoculation.

Inoculation with rhizobia and spontaneous nodulation

M. truncatula chimeric plantlets were transferred in 250 mL containers filled with attapulgite, watered with 20 mL of Farhaeus medium supplemented with 1 mM NH_4NO_3. After 4 days, 2.5 mL of a suspension at OD_{600nm} = 0.025 of a *S. meliloti* strains 2011 harboring the hemA-lacZ plasmid (pXLGD4) was added around the hypocotyl. Roots were harvested, washed and stained 4 weeks post inoculation.

For complementation experiments, *L. japonicus* chimeric plantlets were transferred to Weck jars containing 300 mL of a mix of sand and vermiculite and inoculated with 20 mL of a *M. loti* MAFF303099 *DsRED* suspension in FP medium (OD_{600} = 0.05). Plants were phenotyped 25 days post inoculation.

Spontaneous nodulation experiments on *L. japonicus* roots were performed as described previously [40]. *L. japonicus* chimeric plantlets were transferred to Fahraeus medium plates containing 0.1 μM of the ethylene biosynthesis inhibitor L-α-(2-aminoethoxy-vinyl)-glycine 2.5 weeks after transformation. Root systems were analyzed 60 days post transformation.

Transient Expression in N. benthamiana

Leaves of *N. benthamiana* were infiltrated with *A. tumefaciens* LBA4404 virGN54D strains as described in [79]. Leaves were harvested 3 days after infiltration.

METHOD DETAILS

Microscopy

Tomato ROC expressing SlLYK10-YFP were incubated at room temperature 5 min in water with 1 μg / ml DAPI or 20 μM FM4-64 before confocal imaging. For plasmolysis, ROC lines were incubated for 1 h in 0.8 M mannitol. Tomato ROC and chimeric *M. truncatula* plants expressing the GUS reporter were stained with 0.1% X-Gluc or Magenta-Gluc (20 min under vacuum followed by incubation at 37°C). AMF were stained by treating root tissues with 100% ethanol for 4 h, then with 10% KOH for 8 min at 95°C (tomato ROC and *P. hybrida* roots) or 1,5 days at room temperature (*M. truncatula* roots) and finally with 0.2 M PBS pH 7.2, Triton X-100 0.01%, 1 μg/mL WGA CF488A conjugate overnight at room temperature. For analysis of subcellular localization, tomato ROC and *N. benthamiana* leaves were imaged using a SP8 confocal microscope. Arbuscules in *P. hybrida* were imaged with a SP2 confocal microscope. Overlay corresponds to merge of green fluorescence channel images with differential interference contrast images. GUS and WGA staining were imaged using an Axiozoom V16 microscope (Figure 4) or an Axioplan 2 microscope (Figure 5). Automatic delimitation and drawing of cells strongly expressing GUS was performed with ImageJ (Figure 4).

Numbers of colonization sites and root length colonization were quantified on entire root systems using a S6E microscope after ink staining of the AMF as described in [21].

M. truncatula nodulated roots systems expressing the GUS reporter were stained with 0.1% mangenta-gluc and then fixed with glutaraldehyde 1.25% in 0.1 M PBS pH7.2 (30 min under vacuum). In case of *Mtnfp-2* complementation, nodulated roots systems were first fixed with glutaraldehyde 1.25% and then stained with 2% X-Gal (30min under vacuum and followed by incubation at 28°C). Nodules were sectioned after inclusion in 6% agarose low gelling temperature using a vribratome VT 1000S and sections were imaged with an Axioplan 2 microscope. Nodules of *M. truncatula* roots expressing the GUS reporter under the control of the minimal promoters were stained 0.1% X-Gluc and directly imaged with an Axiozoom V16 microscope.

Western blotting and membrane fraction preparation

Immunobloting of YFP fusions in *M. truncatula* roots was performed on 20 mg of a total extract of a pool of 10 root systems inoculated by *S. meliloti*. For LCO binding assays, approximately 20 g of leaves were homogenized at 4°C in a blender in the presence of 40 mL of extraction buffer (25 mM Tris, pH 8.5, 0.47 M sucrose, 5 mM EDTA, 10 mM DTT, 0.6% PVPP and protease inhibitors (0.1 mM AEBSF, and 1 mg/mL each of leupeptin, aprotinin, antipain, chymostatin, and pepstatin). Samples were centrifuged for 15 min at 3000 *g*, and then the supernatant was recentrifuged for 30 min at 45000 *g*. The pellet (membrane fraction) was first washed in 5 mL and then resuspended in 2 mL of binding buffer (25 mM Na-Cacodylate pH 6, 250 mM sucrose, 1 mM CaCl2, 1 mM MgCl2 and protease inhibitors). After each extraction, amount of fusion proteins was quantified by immunoblotting in 10 μg of membrane

fraction proteins. PhLYK10-YFP, PhLYK10c-YFP and SlLYK10c-YFP have expected molecular masses of about 104, 102 and 102 kDa respectively (including 6 predicted N-glycans). For Figure S4D, after homogenization samples were centrifuged for 20 min at 100000 *g* and resuspended in the same volume of extraction buffer. Proportional volumes of total extract, resuspended pellet and supernatant were loaded on SDS-PAGE.

LCO binding assays

LCO-V(C18:1Δ11,NMe) and LCO-V(C18:1Δ11,NMe,S) were purified from the rhizobial strain *Rhizobium tropici*. Labeling of LCO-V(C18:1Δ11,NMe) was performed as described in [80]. LCO binding assays on membrane fractions containing 20 μg or 40 μg of proteins were performed as in [6] using between 1 and 2 nM of radiolabeled LCO and ranges of unlabeled LCO between 1 nM to 1 μM. Similar amount of membrane fraction from leaves expressing PhLYK10-YFP, PhLYK10-YFPc, SlLYK10c-YFP or from untransformed leaves were used in each experiment. Competition with COs were performed with 1 μM of unlabeled pure CO4 and CO8.

PNGaseF treatment and immunoblotting

PNGaseF treatment, SDS-PAGE, transfer to nitrocellulose membranes and western blotting were performed as described in [30].

qRT-PCR

RNA extraction, cDNA synthesis was performed as described in [21]. Relative expression levels were calculated using glyceraldehyde-3-phosphate dehydrogenase (*GAPDH*) as a reference gene. Primers were as in [81] and [21].

Promoter investigation

MtNFP orthologs were retrieved from genomes of 71 dicotyledonous species (list in Table S2) using tBLASTn and an e-value threshold of $1^{e\text{-}10}$. Putative orthologs were aligned with MAFFT with default parameters and aligned positions with more than 50% of gaps were removed using TrimAl. The best-fitting evolutionary model was tested using ModelFinder and according to the Bayesian Information Criteria. The model TVM+F+R5 was further used for Maximum Likelihood (ML) analysis using IQ-TREE. Branch support was tested using 10,000 replicates of SH-alrt. The resulting tree was annotated using the iTOL platform. For each ortholog, 600 bp promoter sequences were extracted upstream of the gene start using a custom Python script. Promoters were searched for enriched motif using MEME with following parameters: zero or one occurrence of motif per site, motif length comprises between 5 and 25 bp and a minimum of 2 sites by motifs.

QUANTIFICATION AND STATISTICAL ANALYSIS

Number of independent biological replicates and individuals analyzed, as well as the statistical tests used to analyze the data are indicated in the figure legends. All statistical analyses were performed using the R software (http://r-project.org).

DATA AND CODE AVAILABILITY

This study did not generate any unique datasets or code.

Current Biology, Volume *29*

Supplemental Information

LCO Receptors Involved in Arbuscular Mycorrhiza Are Functional for Rhizobia Perception in Legumes

Ariane Girardin, Tongming Wang, Yi Ding, Jean Keller, Luis Buendia, Mégane Gaston, Camille Ribeyre, Virginie Gasciolli, Marie-Christine Auriac, Tatiana Vernié, Abdelhafid Bendahmane, Martina Katharina Ried, Martin Parniske, Patrice Morel, Michiel Vandenbussche, Martine Schorderet, Didier Reinhardt, Pierre-Marc Delaux, Jean-Jacques Bono, and Benoit Lefebvre

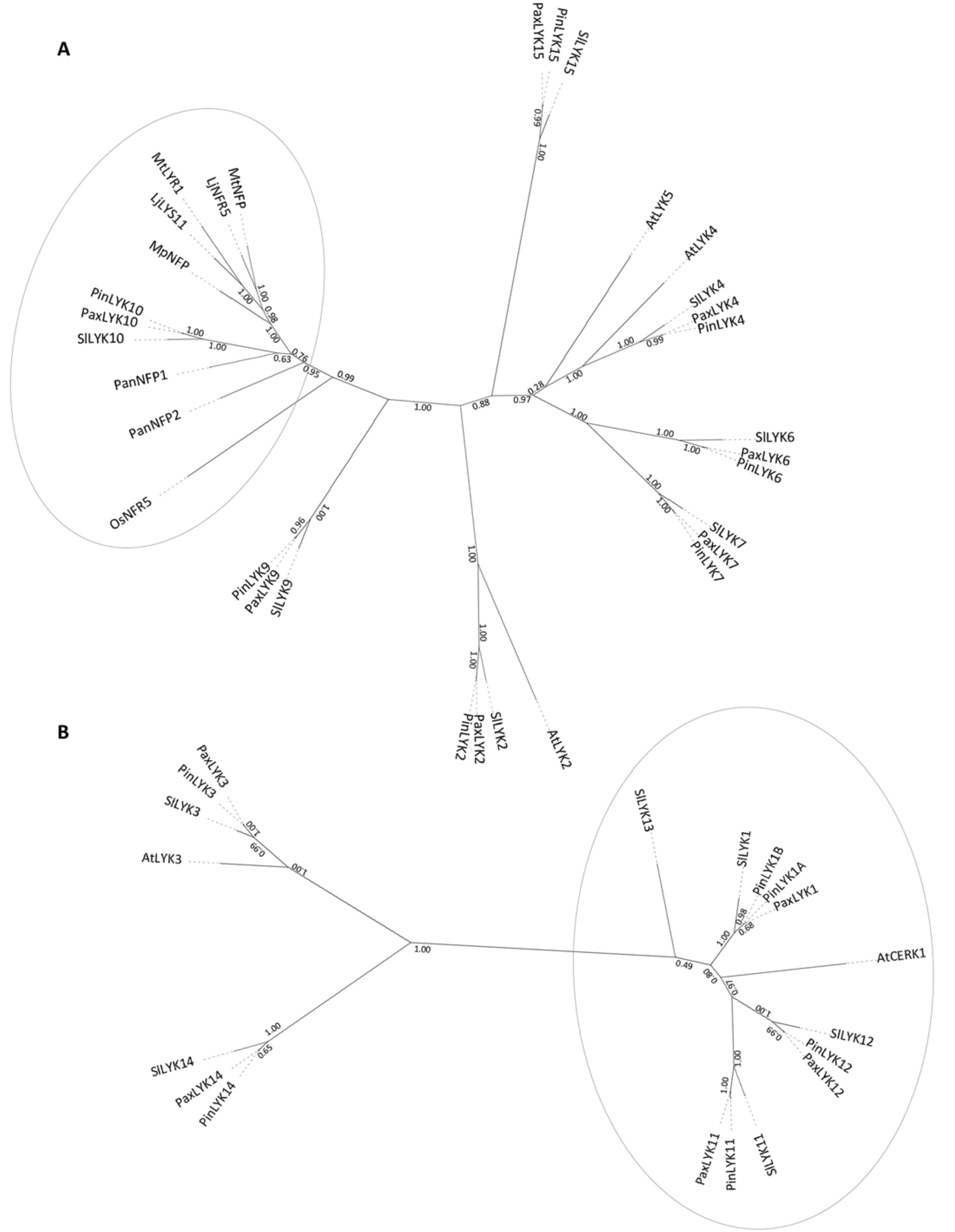

Figure S1 Phylogenetic trees of LYR and LYK LysM-RLKs. Related to Figures 1 and 2. Full length amino acid sequences of all LYRs, predicted to contain inactive kinase domains (A) or LYKs, predicted to contain active kinase domains (B) from *Petunia axillaris* (Pax), *Petunia inflata* (Pin), *Solanum lycopersicum* (Sl) and *Arabidopsis thaliana* (At) were used to construct phylogenetic trees. Accession numbers of *P. axillaris and P. inflata* LysM-RLKs can be found in table S1. Members of the phylogenetic group *LYRIA* (A, circled) from *Medicago truncatula* (Mt), *Lotus japonicus* (Lj), *Mimosa pudica* (Mp), *Parasponia andersonii* (Pan) and *Oryza sativa* (Os) were added. SlLYK8 was not included because it has a truncated kinase. The phylogenetic group *LYKI* containing AtCERK1 and SlLYK12 is circled (B). Numbers at the branches correspond to approximate likelihood values.

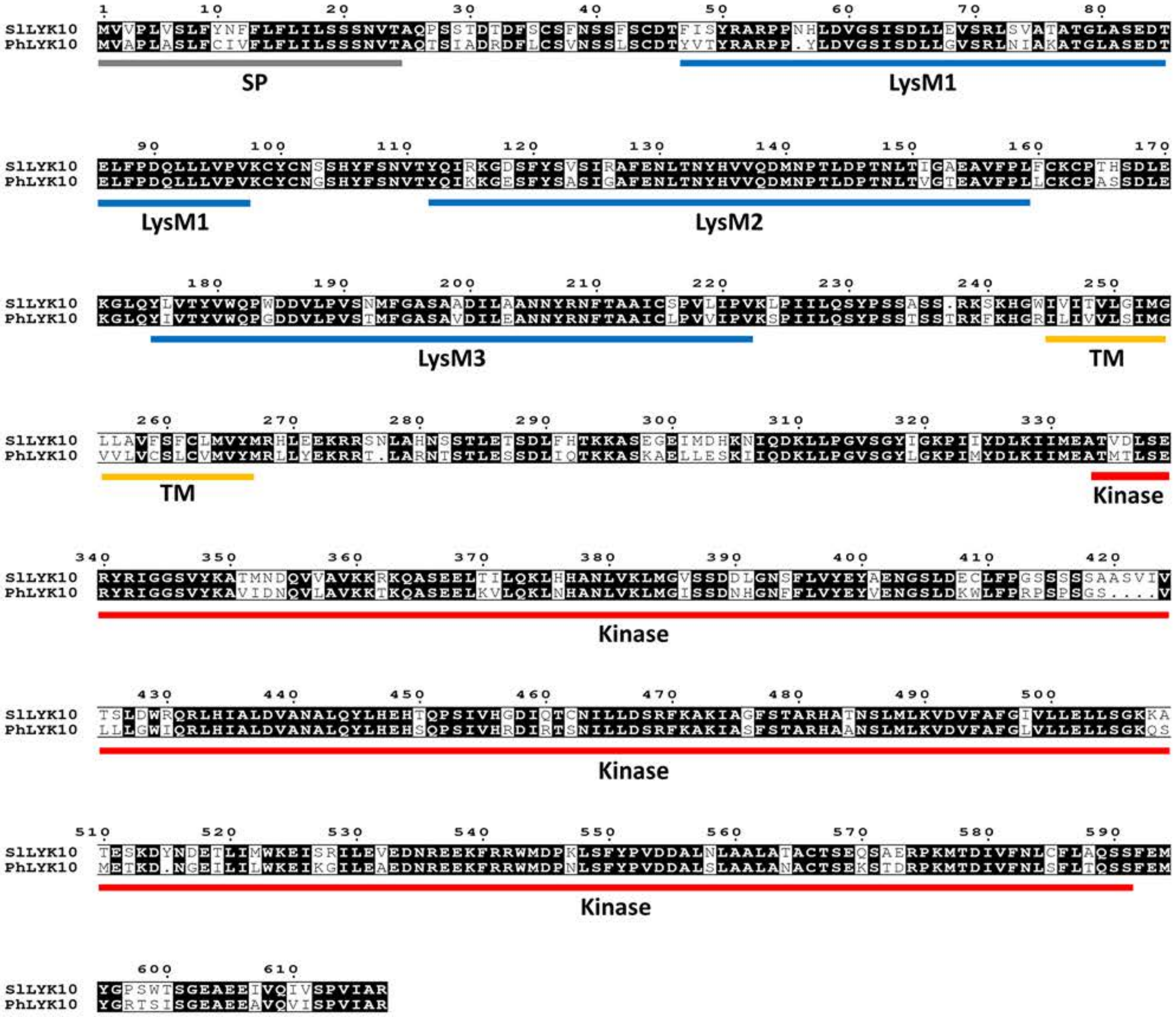

Figure S2 Sequence alignment and annotation of PhLYK10 and SlLYK10. Related to Figures 1 and 2. *PhLYK10* in *Petunia hybrida* originates from the *Petunia axillaris* parent. PhLYK10 has 79% amino acid identity compared to SlLYK10.

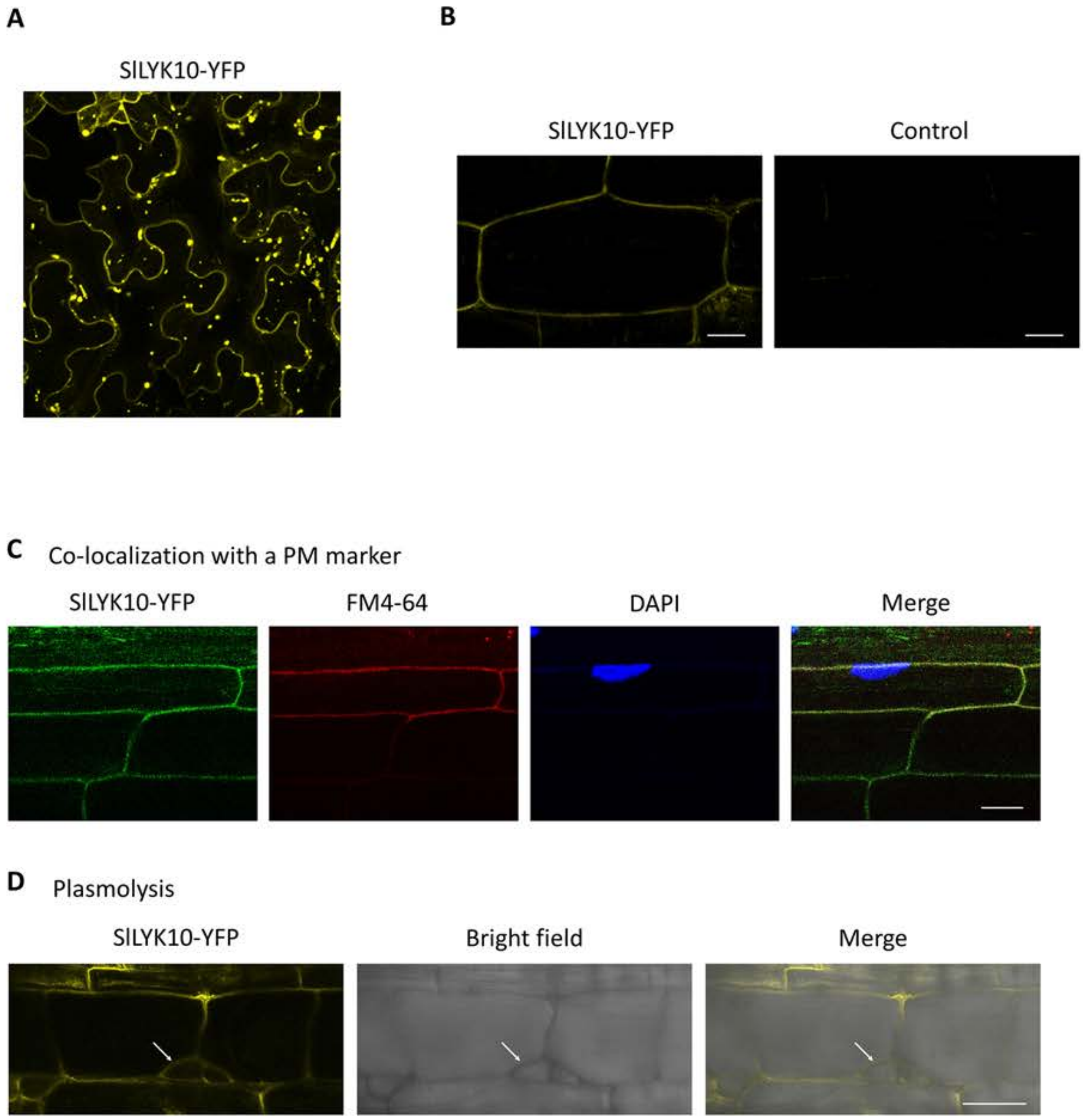

Figure S3 Subcellular localization of SlLYK10-YFP in *N. benthamiana* leaves and tomato roots. **Related to Figure 3.** A) Confocal images of epidermal cells from a *N. benthamiana* leaf expressing *Sl*LYK10-YFP and showing localization partly in undefined compartments. B) Confocal images of epidermal cells from a tomato ROC line expressing SlLYK10-YFP or a ROC control. Images were acquired with similar microscope settings. C) Confocal images of epidermal cells from a tomato ROC line expressing SlLYK10-YFP and treated with FM4-64 (which labels the plasma membrane (PM) before being internalized) and DAPI (which labels the nucleus), showing that SlLYK10-YFP is localized at the PM. D) Confocal images of epidermal cells from a tomato ROC line expressing SlLYK10-YFP and plasmolyzed with mannitol. The arrow points to detachment of the PM from the cell wall. Scale bars represent 20 µm in A, 50 µm in B and 10 µm in C.

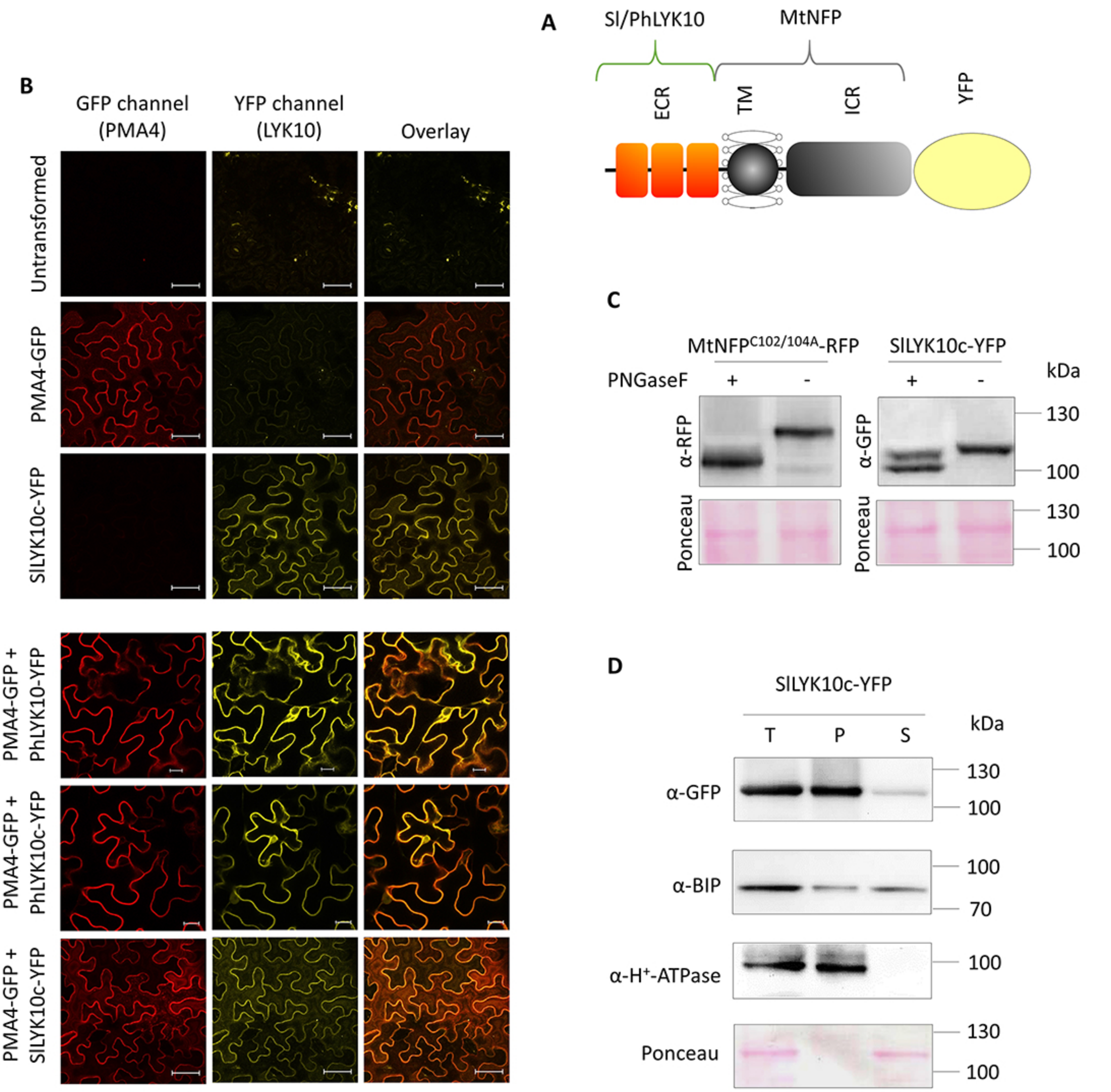

Figure S4 Subcellular localization of SlLYK10c-YFP in *N. benthamiana* leaves. Related to Figure 3. A) Schematic view of the chimeric LYK10c-YFP constructs: the extracellular region (ECR) composed of 3 Lysin Motifs from SlLYK10 or PhLYK10, the transmembrane domain (TM), the intracellular region (ICR) from MtNFP and the yellow fluorescent protein (YFP). B) Co-localization in *N. benthamiana* leaves of PhLYK10-YFP, PhLYK10c-YFP and SlLYK10c-YFP with a plasma membrane (PM) marker. Confocal images of epidermal cells from *N. benthamiana* leaves expressing combinations of PMA4-GFP, a PM marker with PhLYK10-YFP, PhLYK10-YFPc or SlLYK10c-YFP. Fluorescence detected in the GFP and YFP channels is shown in red and yellow respectively, to facilitate visualization of the overlay. Bars represent 20 µm. C) Sensitivity assay of SlLYK10c to PNGaseF. After been processed in the Golgi apparatus, protein N-glycans become insensitive to digestion by PNGaseF. Proteins extracted from *N. benthamiana* leaves were incubated (+) or not (-) with PNGaseF. A higher band after incubation indicates that protein N-glycans are insensitive to the PNGaseF and shows that protein has left the ER. A lower band after incubation indicates that protein N-glycans are sensitive to the PNGaseF digestion and shows that protein did not exit the ER. The mutated form of MtNFP lacking disulfide bridges is retained in the ER [S1] and completely digested by PNGaseF. D) Cell fractionation of leaves expressing SlLYK10c-YFP. Proteins were immunodetected in the total extract (T), the 100,000*g* pellet (P) and the supernatant (S) using anti-GFP antibodies (to detect the YFP fusions), anti-BIP antibodies (to detect an ER marker) or anti-H$^+$-ATPase antibodies (to detect a PM marker). The Ponceau staining shows that equal amounts of proteins were loaded on the gel.

A

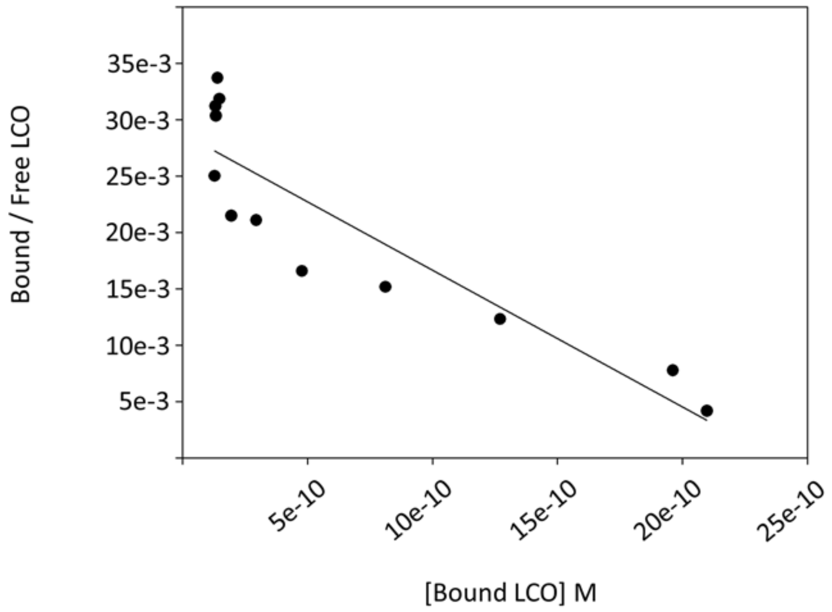

B

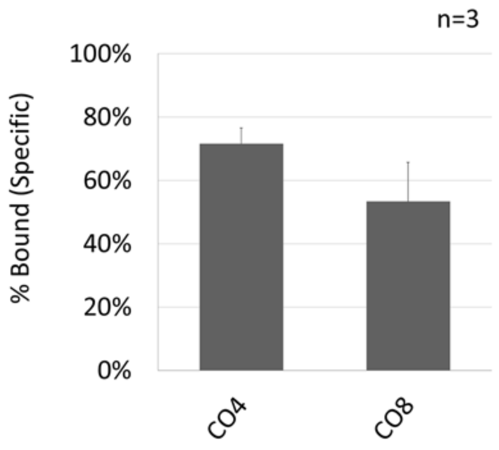

Figure S5 Affinity for LCOs and selectivity for LCOs versus COs of PhLYK10c. **Related to Figure 3.** A) Scatchard plot analysis of a cold saturation experiment using LCO-V(C18:1,NMe,S) and a membrane fraction containing PhLYK10c-YFP. B) Selectivity of the PhLYK10c LCO binding site for various CO structures. Membrane fraction containing PhLYK10c-YFP was incubated with radiolabeled LCO-V(C18:1,NMe,^{35}S) in the presence of 1 µM of the indicated unlabeled COs used as competitors. Non-specific binding was determined in the presence of 1 µM LCO-V(C18:1,NMe,S). Bars represent the percentage of specific binding (means and standard deviations) obtained with independent batches of membrane fractions.

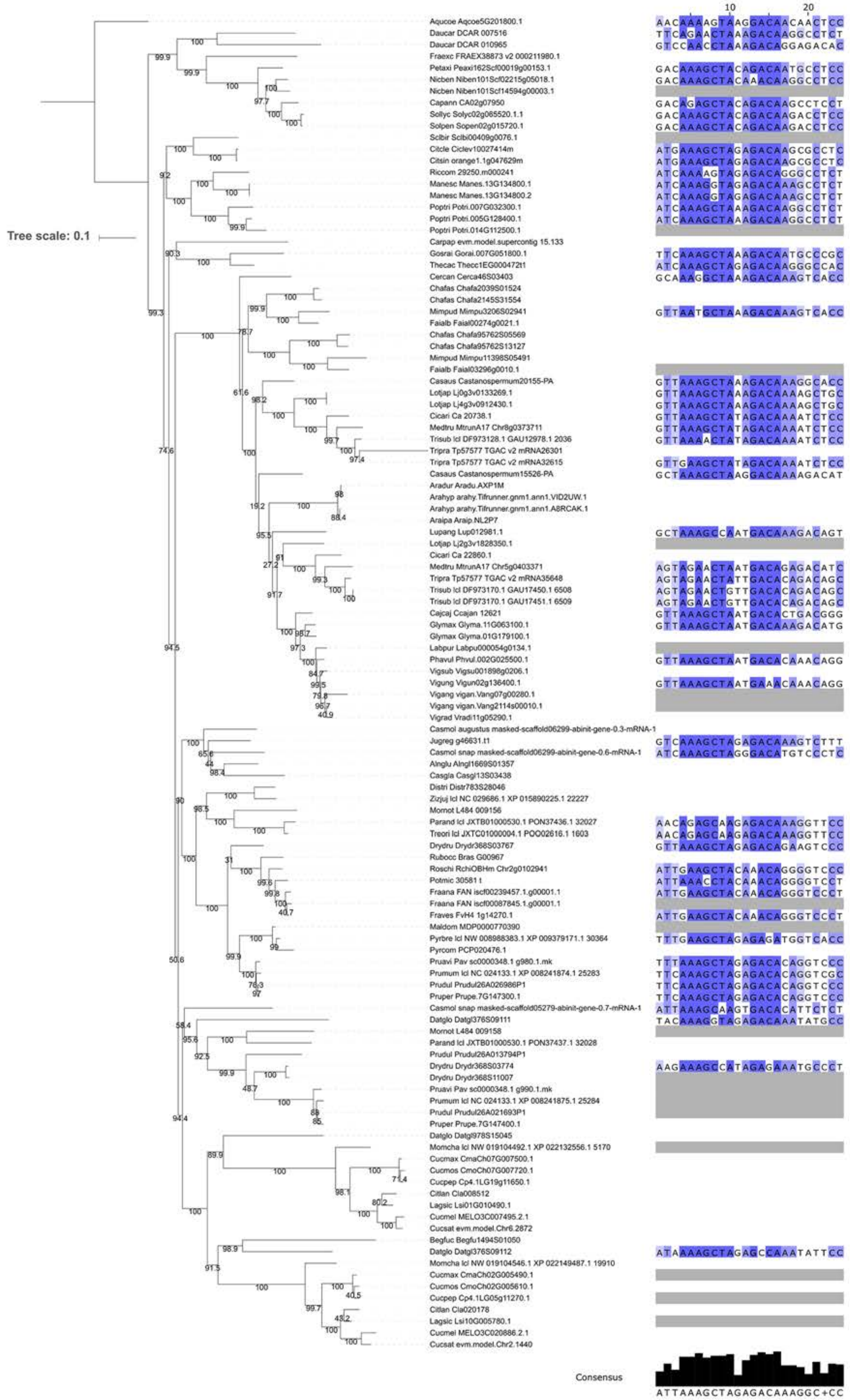

Figure S6 Alignment of the putative *cis*-regulating element in *LYRIA* promoters of 71 dicotyledonous species. **Related to Figure 5.** The tree has been rooted on the basal dicots *Aquilegia coerulea*. Branch support (10,000 replicates of SH-aLRT) is indicated on each branch. Alignment of the putative *cis*-regulating element has been mapped at the right of the tree. Identity percentage is represented by the blue gradient background at each position. Grey and white rectangles indicates that no promoter sequences were extracted for these genes or absence of the motif respectively. Details about the 71 species are given in Table S2.

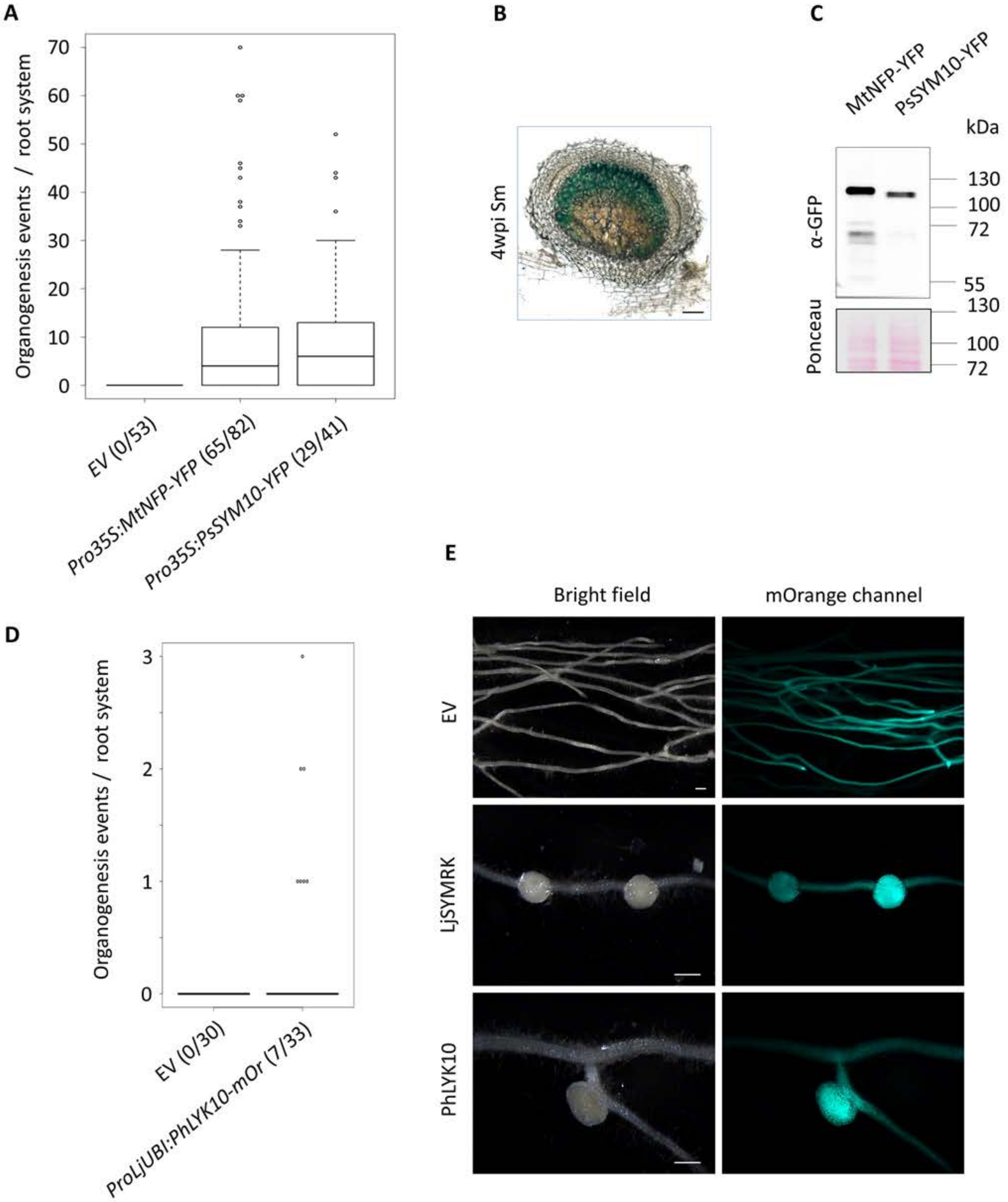

Figure S7 Complementation of *Mtnfp* nodulation by *Pro35S:PsSYM10-YFP* and spontaneous nodulation in *L. japonicus* roots expressing *ProLjUbi:PhLYK10-Orange*. Related to Figure 6. A) Number of organogenesis events (nodules and nodule primordia) 28 dpi with *S. meliloti* lacZ in *Mtnfp* roots transformed with the indicated constructs. Numbers in brackets indicate the numbers of root systems carrying organogenesis events / root systems analyzed. Box plots represent the distribution between individuals from at least 2 independent experiments. Data for the empty vector (EV) and *Pro35:MtNFP-YFP* are the same as in Figure 6A. B) Section of a nodule from roots expressing PsSYM10. *S. meliloti* LacZ were stained by X-Gal. Scale bar represents 100 µm. C) Immunodetection in 20 mg of roots (10 root systems were pooled) expressing the indicated YFP-fusion proteins. D) Roots of *L. japonicus* Gifu wild-type were transformed with the indicated constructs. Box plots represent the number of spontaneous organogenesis events. E) Pictures of spontaneous nodules on *L. japonicus* Gifu roots transformed with EV, *ProLjUBI:LjSYMRK-mOrange* or *ProLjUbi:PhLYK10-mOrange*, 60 days post transformation. Scale bars represent 1 mm.

Subfamily	*Petunia axillaris*	Short name	*Petunia inflata*	Short name	tomato ortholog
**LYR**	Peaxi162Scf00019g00153	PaxLYK10	Peinf101Scf00925g10002	PinLYK10	SlLYK10
	Peaxi162Scf01416g00014	PaxLYK9	Peinf101Scf00428g04012	PinLYK9	SlLYK9
	x		x		SlLYK8
	Peaxi162Scf00420g00445	PaxLYK2	Peinf101Scf01192g06044	PinLYK2	SlLYK2
	Peaxi162Scf00948g00026	PaxLYK15	Peinf101Scf03917g00010	PinLYK15	SlLYK15
	Peaxi162Scf00535g00125*	PaxLYK4	Peinf101Scf00359g10025*	PinLYK4	SlLYK4
	Peaxi162Scf00535g00125*	PaxLYK7	Peinf101Scf00359g10025*	PinLYK7	SlLYK7
	Peaxi162Scf00064g00036	PaxLYK6	Peinf101Scf00471g16004	PinLYK6	SlLYK6
**LYK**	Peaxi162Scf00921g00021	PaxLYK1	Peinf101Scf01180g09048*	PinLYK1A	SlLYK1
	x		Peinf101Scf01180g09048*	PinLYK1B	SlLYK1
	x		x		SlLYK13
	Peaxi162Scf00178g00310*	PaxLYK12	Peinf101Scf00005g00016*	PinLYK12	SlLYK12
	Peaxi162Scf00178g00310*	PaxLYK11	Peinf101Scf00005g00016*	PinLYK11	SlLYK11
	Peaxi162Scf00013g00053	PaxLYK14	Peinf101Scf00500g06038	PinLYK14	SlLYK14
	Peaxi162Scf00038g01033	PaxLYK3	Peinf101Scf02312g02018	PinLYK3	SlLYK3

Table S1 LysM-RLKs in *Petunia axillaris* and *Petunia inflata* genomes. Related to Figure 2.
* Genes in tandem for which the gene model predicts only one gene.

Species	Order	Family	Abbreviation	Reference	RNS	AM
Alnus glutinosa	Fagales	Betulaceae	Alnglu	10.1126/science.aat1743	1	1
Aquilegia coerulea	Ranunculales	Ranunculaceae	Aqucoe	10.7554/eLife.36426	0	ND
Arachis duranensis	Fabales	Fabaceae	Aradur	10.1038/ng.3517	1	1
Arachis hypogaea	Fabales	Fabaceae	Arahyp	peanutbase.org	1	1
Arachis ipaensis	Fabales	Fabaceae	Araipa	10.1038/ng.3517	1	1
Begonia fuchsioides	Cucurbitales	Begoniaceae	Begfuc	10.1126/science.aat1743	0	1
Cajanus cajan	Fabales	Fabaceae	Cajcaj	10.1038/nbt.2022.	1	1
Capsicum annuum cvCM334	Solanales	Solanaceae	Capann	10.1038/ng.2877	0	1
Carica papaya	Brassicales	Caricaceae	Carpap	10.1038/nature06856	0	1
Castanea mollissima	Fagales	Fagaceae	Casmol	www.hardwoodgenomics.org	0	1
Castanospermum australe	Fabales	Fabaceae	Casaus	10.1126/science.aat1743	0	1
Casuarina glauca	Fagales	Casuarinaceae	Casgla	10.1126/science.aat1743	1	1
Cercis canadensis	Fabales	Caesalpiniaceae	Cercan	10.1126/science.aat1743	0	1
Chamaecrista fasciculata	Fabales	Fabaceae	Chafas	10.1126/science.aat1743	1	1
Cicer arietinum ICC4958	Fabales	Fabaceae	Cicari	10.1038/srep12806	1	1
Citrullus lanatus subsp. vulgaris 97103	Cucurbitales	Cucurbitaceae	Citlan	10.1038/ng.2470	0	1
Citrus clementina	Sapindales	Rutaceae	Citcle	10.1038/nbt.2906	0	1
Citrus sinensis	Sapindales	Rutaceae	Citsin	10.1038/nbt.2906	0	1
Cucumis melo	Cucurbitales	Cucurbitaceae	Cucmel	10.1073/pnas.1205415109	0	1
Cucumis sativus PI183967	Cucurbitales	Cucurbitaceae	Cucsat	10.1038/ng.2801	0	1
Cucurbita maxima	Cucurbitales	Cucurbitaceae	Cucmax	10.1016/j.molp.2017.09.003	0	1
Cucurbita moschata	Cucurbitales	Cucurbitaceae	Cucmos	10.1016/j.molp.2017.09.004	0	1
Cucurbita pepo	Cucurbitales	Cucurbitaceae	Cucpep	http://cucurbitgenomics.org	0	1
Datisca glomerata	Cucurbitales	Dasticaceae	Datglo	10.1126/science.aat1743	1	1
Daucus carota	Apiales	Apiaceae	Daucar	10.1038/ng.3565	0	1
Discaria trinervis	Rosales	Rhamnaceae	Distri	10.1126/science.aat1743	1	1
Dryas drummondii	Rosales	Rosaceae	Drydru	10.1126/science.aat1743	1	1
Faidherbia albida	Fabales	Fabaceae	Faialb	10.1093/gigascience/giy152	1	1
Fragaria vesca	Rosales	Rosaceae	Fraves	10.1093/gigascience/gix124	0	1
Fragaria x ananassa	Rosales	Rosaceae	Fraana	www.rosaceae.org	0	1
Fraxinus excelsior	Lamiales	Oleaceae	Fraexc	10.1038/nature20786	0	1
Glycine max	Fabales	Fabaceae	Glymax	10.1038/nature08670	1	1
Gossypium raimondii	Malvales	Malvaceae	Gosrai	10.1038/nature11798	0	1
Juglans regia	Fagales	Juglandaceae	Jugreg	10.1111/tpj.13207	0	1
Lablab purpureus	Fabales	Fabaceae	Labpur	10.1093/gigascience/giy152	1	1
Lagenaria siceraria	Cucurbitales	Cucurbitaceae	Lagsic	10.1111/tpj.13722	0	1
Lotus japonicus	Fabales	Fabaceae	Lotjap	10.1093/dnares/dsn008	1	1
Lupinus angustifolius	Fabales	Fabaceae	Lupang	10.1111/pbi.12615	1	0
Malus domestica	Rosales	Rosaceae	Maldom	www.rosaceae.org	0	1
Manihot esculenta	Malpighiales	Euphorbiaceae	Manesc	10.1038/nbt.3535	0	1
Medicago truncatula	Fabales	Fabaceae	Medtru	10.1038/s41477-018-0286-7	1	1
Mimosa pudica	Fabales	Fabaceae	Mimpud	10.1126/science.aat1743	1	1
Momordica charantia	Cucurbitales	Cucurbitaceae	Momcha	NCBI	0	1
Morus notabilis	Rosales	Moraceae	Mornot	10.1038/ncomms3445	0	1
Nicotiana benthamiana	Solanales	Solanaceae	Nicben	10.1094/MPMI-06-12-0148-TA	0	1
Parasponia andersonii	Rosales	Cannabaceae	Parand	10.1073/pnas.1721395115	1	1
Petunia axillaris	Solanales	Solanaceae	Petaxi	10.1038/nplants.2016.74	0	1
Phaseolus vulgaris	Fabales	Fabaceae	Phavul	10.1038/ng.3008	1	1
Populus trichocarpa	Malpighiales	Salicaceae	Poptri	10.1126/science.1128691	0	1
Potentilla micrantha	Rosales	Rosaceae	Potmic	10.1093/gigascience/giy010	0	1
Prunus avium	Rosales	Rosaceae	Pruavi	10.1093/dnares/dsx020	0	1
Prunus dulcis	Rosales	Rosaceae	Prudul	www.rosaceae.org	0	1
Prunus mume	Rosales	Rosaceae	Prumum	NCBI	0	1
Prunus persica	Rosales	Rosaceae	Pruper	10.1038/ng.2586	0	1
Pyrus communis	Rosales	Rosaceae	Pyrcom	10.1371/journal.pone.0092644	0	1
Pyrus x bretschneideri	Rosales	Rosaceae	Pyrbre	NCBI	0	1
Ricinus communis	Malpighiales	Euphorbiaceae	Riccom	10.1038/nbt.1674	0	1
Rosa chinensis	Rosales	Rosaceae	Roschi	10.1038/s41588-018-0110-3	0	1
Rubus occidentalis	Rosales	Rosaceae	Rubocc	10.1111/tpj.13215	0	1
Sclerocarya birrea	Sapindales	Anacardiaceae	Sclbir	10.1093/gigascience/giy152	0	1
Solanum lycopersicum	Solanales	Solanaceae	Sollyc	10.1038/nature11119	0	1
Solanum pennellii	Solanales	Solanaceae	Solpen	10.1038/ng.3046	0	1
Theobroma cacao	Malvales	Malvaceae	Thecac	10.1186/gb-2013-14-6-r53	0	1
Trema orientalis	Rosales	Cannabaceae	Treori	10.1073/pnas.1721395115	0	1
Trifolium pratense	Fabales	Fabaceae	Tripra	10.1038/srep17394	1	1
Trifolium subterraneum	Fabales	Fabaceae	Trisub	NCBI	1	1
Vigna angularis	Fabales	Fabaceae	Vigang	10.1038/srep080669	1	1
Vigna radiata	Fabales	Fabaceae	Vigrad	10.1038/ncomms6443	1	1
Vigna subterranea	Fabales	Fabaceae	Vigsub	10.1093/gigascience/giy152	1	1
Vigna unguiculata	Fabales	Fabaceae	Vigung	Phytozome	1	1
Ziziphus jujuba cv. Dongzao	Rosales	Rhamnaceae	Zizjuj	10.1038/ncomms6315	0	1

Table S2 List of investigated species for promoter analysis. Related to Figure 5.

Supplemental reference

S1: Lefebvre, B., Klaus-Heisen, D., Pietraszewska-Bogiel, A., Herve, C., Camut, S., Auriac, M.-C., Gasciolli, V., Nurisso, A., Gadella, T.W.J., and Cullimore, J. (2012). Role of N-Glycosylation Sites and CXC Motifs in Trafficking of Medicago truncatula Nod Factor Perception Protein to Plasma Membrane. J Biol Chem *287*, 10812-10823.
